# Management of Lymphomas: Consensus Document 2018 by an Indian Expert Group

**DOI:** 10.1007/s12288-018-0991-4

**Published:** 2018-08-03

**Authors:** Reena Nair, Abhishek Kakroo, Ajay Bapna, Ajay Gogia, Amish Vora, Anand Pathak, Anu Korula, Anupam Chakrapani, Dinesh Doval, Gaurav Prakash, Ghanashyam Biswas, Hari Menon, Maitreyee Bhattacharya, Mammen Chandy, Mayur Parihar, M. Vamshi Krishna, Neeraj Arora, Nikhil Gadhyalpatil, Pankaj Malhotra, Prasad Narayanan, Rekha Nair, Rimpa Basu, Sandip Shah, Saurabh Bhave, Shailesh Bondarde, Shilpa Bhartiya, Soniya Nityanand, Sumeet Gujral, T. V. S. Tilak, Vivek Radhakrishnan

**Affiliations:** 1 Department of Clinical Hematology, Tata Medical Center (TMC), New Town, Rajarhat, Kolkata, West Bengal 700 160 India; 2 Vedant Institute of Medical Sciences, Ahmedabad, India; 3 Bhagwan Mahavir Cancer Hospital Research Center (BMCHRC), Jaipur, India; 4 All India Institute of Medical Sciences (AIIMS), New Delhi, India; 5 Pratiksha Hospital, Gurgaon, India; 6 National Cancer Institute (NCI), Nagpur, India; 7 Christian Medical College (CMC), Vellore, India; 8 Apollo Gleneagles Hospital, Kolkata, India; 9 Rajiv Gandhi Cancer Institute and Research Centre (RGCI), New Delhi, Delhi India; 10 Post Graduate Institute of Medical Education and Research (PGIMER), Chandigarh, India; 11 Sparsh Hospital American Oncology Institute (AOI), Bhubaneswar, India; 12 Cytecare Cancer Hospitals, Bangalore, India; 13 Calcutta Medical College, Kolkata, India; 14 Apollo Hospital, Hyderabad, India; 15 Yashoda Hospitals (Somajiguda), Hyderabad, India; 16 Regional Cancer Centre (RCC), Thiruvananthapuram, India; 17 Shatabdi Super Speciality Hospital, Nasik, India; 18 Sanjay Gandhi Post Graduate Institute of Medical Sciences (SGPGIMS), Lucknow, India; 19 Tata Memorial Hospital, Mumbai, India; 20 Command Hospital, Air Force Bangalore, Bangalore, India

**Keywords:** Lymphoma, Consensus statement, Management, Common regimens

## Abstract

The clinical course of lymphoma depends on the indolent or aggressive nature of the disease. Hence, the optimal management of lymphoma needs a correct diagnosis and classification as B cell, T-cell or natural killer (NK)/T-cell as well as indolent or high-grade type lymphoma. The current consensus statement, developed by experts in the field across India, is intended to help healthcare professionals manage lymphomas in adults over 18 years of age. However, it should be noted that the information provided may not be appropriate to all patients and individual patient circumstances may dictate alternative approaches. The consensus statement discusses the diagnosis, staging and prognosis applicable to all subtypes of lymphoma, and detailed treatment regimens for specific entities of lymphoma including diffuse large B-cell lymphoma, Hodgkin’s lymphoma, follicular lymphoma, T-cell lymphoma, chronic lymphocytic leukemia/small lymphocytic lymphoma, Burkitt’s lymphoma, and anaplastic large cell lymphoma.

## Introduction

Lymphomas are a heterogenous group of lymphoproliferative disorders, which are broadly classified as Hodgkin’s lymphoma (HL) and non-Hodgkin’s lymphoma (NHL). The lymphomas arise from B-cell, T-cell and natural killer (NK)/T-cell lymphocytes. B-cell lymphomas account for 80–85% of all NHLs and T-cell and NK/T-cell lymphomas account for the remaining 15–20% [1–3].

The clinical course depends on the indolent or aggressive nature of the lymphoma. While aggressive high-grade lymphomas are generally curable with cytotoxic therapies, indolent lymphomas are controllable for long periods with minimal cytotoxic therapy. Hence, it is not only imperative to make a correct diagnosis of lymphoma, but it is equally important to correctly classify them as B- cell, T-cell or NK/T-cell as well as indolent or high-grade, for optimal management.

Indian Council of Medical Research (ICMR) published a consensus statement in 2017 [1] on the management of aggressive lymphomas. Since then, there have been major changes in the classification of lymphomas, as well as the availability of new therapies to treat lymphomas that relapse. This consensus statement has included the changes in the management of all major subtypes of lymphomas.

## Objectives

The objective of this consensus statement is to provide healthcare professionals with current information on the management of lymphomas in patients above 18 years of age. However, it should be noted that the information provided may not be appropriate to all patients and individual patient circumstances may dictate alternative approaches.

The collaborative nature of this consensus statement hopes to emphasize and nurture the need for more such efforts at the national platform in India. An ongoing lymphoma registry program is attempting to capture information on the demographics and outcomes of patients with lymphoma, and many of the participants in this consensus document are members of this volunteer registry. Much more, however, needs to be done on collaborative projects in lymphoma and other cancers at the national and regional platforms.

Following some general comments regarding diagnosis, staging and prognosis applicable to all subtypes of lymphoma, the consensus document discusses in more detail the therapies in relation to specific entities of lymphoma as defined in the World Health Organization (WHO) [4] classification, which include mature B-cell neoplasms, mature T-cell and NK-cell neoplasms, HL, histiocytic and dendritic cell neoplasms and post-transplant lymphoproliferative disorders. In adults, HL, diffuse large B-cell lymphoma (DLBCL), follicular lymphoma (FL), lymphoblastic lymphoma (LBL), small lymphocytic lymphoma (SLL), Burkitt’s lymphoma (BL), Peripheral T cell lymphoma (PTCL), anaplastic large cell lymphoma (ALCL), etc. are the most common types [1].

## Diagnostic Biopsy: Points to Remember [5, 6]

Excision biopsy of the most prominent and accessible largest palpable lymph node should be considered first.

1. When the aforementioned is not possible, a Needle Core Biopsy (NCB) should be advocated with at least 4–5 cores. The NCB can be considered for sites that are difficult to access such as lung, mediastinum, abdomen, retroperitoneum etc. In exceptional circumstances, NCB may also be done in palpable lumps such as in the elderly or severely ill patients. The NCB procedure demands expert radiologists. In patients where NCB or fine-needle aspiration cytology (FNAC) can’t be performed, thoracotomy or laparotomy can be considered to obtain adequate tissue to facilitate the diagnosis. Management based on a FNAC diagnosis alone should be avoided as it has limitations. Steroid use has to be restricted, if possible, until diagnostic material is collected, as it may cause remissions in patients with very sensitive disease and delay the diagnosis.
2. The FNAC and body fluids may be sent for flow cytometric immuno-phenotyping (FCI). The laboratory should have standard operating procedures (SOPs) to perform FCI. The prepared slides should also be sent for morphological evaluation.
3. Blunt needles need to be avoided as they cause crushing artifacts, limiting morphological interpretations.
4. The laboratory should have extensive immunohistochemistry (IHC) markers panel. The minimum panel for each type of lymphoma has to be defined in the SOPs. A comprehensive IHC work up is advisable. In resource challenged situations, a practical and validated working algorithm is encouraged. In difficult cases, a second opinion may be taken from an expert lymphoma pathologist. For a specialist hemato-pathologist opinion, referral laboratories should be well defined and documented. Similarly, referral laboratories need to be defined and documented for FCI, fluorescence in situ hybridisation (FISH) and molecular tests.

## Essential Evaluation and Staging Work-Up

### Staging Work-Up: All Patients [7]

#### Mandatory Clinical History and Examination

1. Clinical history with reference to B symptoms
2. Physical examination with particular attention to node-bearing areas, waldeyer’s ring, liver span, splenic enlargement, and testicular enlargement (in males).
3. Performance status (Eastern Cooperative Oncology Group; ECOG) including co-morbidity
4. Need to watch for features of an “oncological emergency” such as: tumor lysis syndrome, spinal cord compression, luminal obstruction, raised intra-cranial pressures due to mass effect, pericardial tamponade, etc.

#### Mandatory Staging Procedure

1. Complete blood count (CBC) inclusive of differential counts, peripheral blood film and erythrocyte sedimentation rate (ESR) for early stage HL
2. Bone marrow aspirate and trephine biopsy (a unilateral biopsy is sufficient if biopsy material is adequate and > 1.5 cm in length), flow cytometry for chronic lymphoproliferative disorders (CLPDs), if indicated
3. Lactate dehydrogenase (LDH), creatinine, uric acid, urea and electrolytes, S-proteins, aspartate transaminase (AST), bilirubin, alkaline phosphatase, and calcium
4. Pregnancy test in females of child-bearing age
5. Hepatitis B and C, human immunodeficiency virus (HIV) status, hepatitis B core antigen (HBcAg) must be done prior to initiating chemo/immunotherapy
6. Chest and abdomino-pelvic computed tomography (CT) with oral and intravenous (IV) contrast (unless coexistent renal insufficiency). Integrated positron emission tomography–computed tomography (PET-CT) has largely replaced the CT scan.
7. In a resource challenged setting: chest X-ray and abdominal ultrasonography (USG)

### Staging Work-Up: Indicated in Special Conditions [8–10]

1. Full coagulation profile
2. Direct Coombs Test (DCT); especially in low grade lymphomas and chronic lymphocytic leukemia [CLL]), and reticulocyte count
3. Cytogenetics and immunophenotyping of marrow ± blood in low grade lymphomas and any other lymphomas with morphological evidence of marrow/blood involvement
4. If there is lymphocytosis, consider peripheral blood FCI (especially in low grade lymphomas/CLL)
5. Serum protein electrophoresis and quantitative IgG and IgM for indolent B-cell lymphomas
6. Β-2 microglobulin
7. Epstein-Barr virus (EBV), human T-cell lymphotropic virus (HTLV) serology
8. *H. pylori* serology (gastric lymphoma)

#### Molecular Genetics [5, 6]

1. FISH or polymerase chain reaction (PCR) on involved marrow/blood for specific lymphoma-associated translocations
2. Immunoglobulin heavy chain (IgH) and T cell receptor (TCR) rearrangements on marrow/blood if molecular staging is clinically indicated

#### Radiology [5, 6]

1. Plain bone X-ray and bone scintigraphy skeletal survey for extranodal bone NHL
2. Magnetic resonance imaging (MRI) or CT scan of the brain, contrast enhanced imaging, when indicated by CNS symptoms and signs

#### Other Important Considerations [5, 6]

1. Multigated acquisition (MUGA) scan or echocardiography (ECG) is recommended when anthracycline containing regimens are used
2. Pulmonary function tests (PFTs) are recommended when bleomycin is contemplated as in HL.
3. Endoscopy and endoscopic ultrasound, head CT scan, or brain MRI and lumbar puncture depending on suspicion of extranodal involvement (Table 1).

**Table:** **Table 1** Resource stratified diagnostic work-up for lymphoma at presentation

Diagnostic Work-up and Staging	Basic	Limited	Enhanced	State-of-the-art
Biopsy—excision/incision/needle core	Morphology	Limited panel IHC to differentiate B and T/NK cell	Extended panel IHC to diagnose and subtype FISH-confirm translocations	Sequencing to detect cell of origin, clonality studies
Clinical examination Chest scanning Abdomen scanning	Physical examination X-ray chest Sonography	CT Scan Neck, Thorax, and Whole Abdomen	PET-CT scan whole body	
Bone Marrow	Aspirate and biopsy	Flow cytometry	Cytogentics and FISH, if indicated	
Extra nodal Imaging	X-rays, sonography	CT scan, Bone scan	MRI, PET-CT scan	

*CT* computed tomography, *FISH* fluorescent in situ hybridization, *IHC* immunohistochemistry, *MRI* magnetic resonance imaging, *PET-CT* positron emission tomography-computed tomography

### Staging of Lymphoma

The optimal management and prognosis of lymphoma depends, in part, on the stage of the lymphoma. The staging system used for adult high grade lymphomas is based on the Ann Abor system (Table 2) [11].

**Table:** **Table 2** Ann Arbor staging for lymphoma

Stage	Area of involvement
I	One lymph node region
IE	One extralymphatic (E) organ or site
II	Two or more lymph node regions on the same side of the diaphragm
IIE	One extralymphatic organ or site (localized) in addition to criteria for stage II
III	Lymph node regions on both sides of the diaphragm
IIIE	One extralymphatic organ or site (localized) in addition to criteria for stage III
IIIS	Spleen (S) in addition to criteria for stage III
IIISE	Spleen and one extralymphatic organ or site (localized) in addition to criteria for stage III
IV	One or more extralymphatic organs with or without associated lymph node involvement (diffuse or disseminated); involved organs should be designated by subscript letters (P, lung; H, liver; M, bone marrow)

Each stage is subdivided into A and B categories; B for those with defined general symptoms (unexplained fever of ≥ 38 °C; unexplained drenching night sweats; or loss of > 10% body weight within the previous 6 months) and A for those without

X = Bulky tumor is defined as either a single mass of tumor tissue exceeding 10 cms in largest diameter or a mediastinal mass exceeding 1/3 of the transverse maximal transthoracic diameter

An international working group incorporated the PET scan and revised the staging criteria [12], which were widely adopted. The 2011 International Conference on Malignant Lymphoma (ICML) in Lugano proposed a revised staging system for primary nodal lymphomas (Table 3) [13, 14].

**Table:** **Table 3** Lugano revised staging system 2014 for primary nodal lymphomas

Stage	Involvement	Extranodal [E] status
*Limited*		
I II	One or a group of adjacent nodes Two or more nodal groups on the same side of the diaphragm	Single extranodal region without nodal involvement Stage I or II by nodal extent with limited contiguous extranodal involvement
II Bulky	II as above with “bulky disease’	Not applicable
*Advanced*		
III IV	Nodes on both sides of the diaphragm; nodes above the diaphragm with spleen involvement Additional non-contiguous extralymphatic involvement	Not applicable Not applicable

Suffix A (asymptomatic) or B (symptomatic) included for HL only

Bone marrow biopsy not indicated for HL and most DLBCL’s

For clinical staging of chronic lymphocytic leukemia (CLL), Rai et al. [15], and Binet et al. [16], proposed criteria, which are based on the concept that CLL is a disease of progressive accumulation of non-functioning lymphocytes (Table 4) [15, 16].

**Table:** **Table 4** Rai and Binet staging criteria for CLL

Stage	Risk	Clinical features
*Rai’s staging for CLL*		
0	Low	Lymphocytosis
I/II	Intermediate	Lymphadenopathy ± hepatosplenomegaly
III/IV	High	Anemia ± thrombocytopenia
*Binet’s staging for CLL*		
A	Low	Lymphocytosis with < 3 areas of adenopathy
B	Intermediate	Lymphocytosis with > 3 areas of adenopathy
C	High	Anemia, thrombocytopenia or both

*CLL* chronic lymphocytic leukemia

## Prognostication

The ECOG performance status (published by Oken et al. in 1982) [17], also called the WHO or Zubrod score (after C. Gordon Zubrod), is a numbering scale used to determine whether the patients can receive chemotherapy, if dose adjustment is necessary, as a measure for the required intensity of palliative care, and as a measure of quality of life in randomized controlled trials (RCTs) (Table 5) [17].

**Table:** **Table 5** Performance index—ECOG performance status

Grade	ECOG performance status
0	Fully active, able to carry on all pre-disease performance without restriction
1	Restricted in physically strenuous activity, but ambulatory and able to carry out work of a light or sedentary nature, e.g., light house work, office work
2	Ambulatory and capable of all self-care but unable to carry out any work activities. Up and about > 50% of waking hours
3	Capable of only limited self-care, confined to bed or chair more than 50% of waking hours
4	Completely disabled, and cannot carry out any self-care. Totally confined to bed or chair

*ECOG* Eastern Cooperative Oncology Group

### Chronic Lymphocytic Leukemia-International Prognostic Index (CLL-IPI)

The CLL-International Prognostic Index (CLL-IPI) is a revised staging system that combines genetic, biochemical, and clinical parameters for a more targeted treatment of CLL. The IPI is a prognostic model based on 5 parameters (Table 6) [18].

**Table:** **Table 6** CLL-international prognostic index

Variables	Risk score
Age (> 65 years)	1
Stage-Rai’s III/IV or Binet B/C	1
del 17p and/or TP 53 mutation	4
IGVH (immunoglobulin heavy chain variable region) unmutated	2
β-2 microglobulin > 3.5 mg/L	2

*CLL* chronic lymphocytic leukemia

Based on these factors, patients with CLL can be divided into 4 prognostic categories as summarized in Table 7 [18, 19].

**Table:** **Table 7** CLL risk classification based on IPI score

IPI risk group	IPI score	5 year overall survival (%)
Low-risk	0–1	93.2
Intermediate-risk	2–3	79.3
High-risk	4–6	63.3
Very High risk	7–10	23.3

*CLL* chronic lymphocytic leukemia, *IPI* international prognostic index

### International Prognostic Index

The Ann Arbor classification does not consistently distinguish between patients with different long-term prognoses; hence, the International Non-Hogdkin’s Lymphoma Prognostic Factor Project provided the international index and age-adjusted international index for the selection of appropriate therapeutic approaches for individual patients [20]. The IPI is a prognostic model based on 5 parameters (Table 8).

**Table:** **Table 8** International prognostic index

Score	0	1
Age (years)	< 60	≥ 60
Performance status	0–1	2–4
Stage	I–II	III–IV
LDH	Normal level	≥ Normal levels
Extranodal sites	≤ 1	> 1

*LDH* lactate dehydrogenase

Based on these factors, patients with DLBCL can be divided into 4 prognostic categories as summarised in Table 9 [20].

**Table:** **Table 9** Classification of DLBCL patients based on IPI scores

IPI risk group	IPI Score	CR Rate (%)	5 year OS (%)
Low-risk	0, 1	87	73
Low/intermediate-risk	2	67	51
High/intermediate-risk	3	55	43
High risk	4, 5	44	26

*DLBCL* diffuse large B-cell lymphoma, *IPI* international prognostic index, *CR* complete response, *OS* overall survival

### Age-Adjusted International Prognostic Index (aa-IPI)

Risk factors for age-adjusted IPI (aa-IPI) include ECOG performance status ≥ 2, Stage III/IV, and LDH greater than the upper limit of normal (ULN) (Table 10). [21]

**Table:** **Table 10** Classification of age-adjusted international prognostic index risk groups

aa-IPI risk group	aa-IPI Score	5 year OS (%)
Low-risk	0	83
Low/intermediate-risk	1	69
High/intermediate-risk	2	46
High risk	3	32

aa-*IPI* age-adjusted international prognostic index

### Revised International Prognostic Index (R-IPI)

In the rituximab era, the IPI has been revised and the patients are grouped as shown in Table 11. The revised IPI (R-IPI) is a better predictor of outcome than the standard IPI for patients with DLBCL treated with R-CHOP (rituximab, cyclophosphamide, doxorubicin, vincristine, and prednisone) [21].

**Table:** **Table 11** Revised international prognostic index

Number of IPI factors	Risk groups	Overall survival (%)
0	Very Good	94
1–2	Good	79
3, 4, 5	Poor	55

*IPI* international prognostic index

The IPI is less useful in ALCL, mediastinal B cell lymphoma and mature T-cell lymphomas. It should not be used for BL and LBL. The IPI has been adjusted for use in FL. The Follicular Lymphoma International Prognostic Index (FLIPI) predicts survival for FL and is used for other indolent lymphomas as well (Table 12) [22].

**Table:** **Table 12** Follicular lymphoma international prognostic index (FLIPI)-1 index

Factor	Adverse	Prognosis	No. of factors	10 years OS (%)
Nodal sites	> 4	Good	0–1	71
LDH	> normal			
Age	> 60	Intermediate	2	51
Ann Arbor stage	III–IV			
Hemoglobin	< 12 gm/dL	Poor	3–5	36

*LDH* lactate dehydrogenase, *OS* overall survival

### Mantle Cell: International Prognostic Score (MIPI)

The Mantle Cell Lymphoma International Prognostic Index (MIPI) shown in Table 13 is superior to the IPI in predicting survival following intensive first-line immunochemotherapy and autologous stem cell transplantation.

**Table:** **Table 13** Mantle cell—international prognostic score (MIPI)

Points	Age (years)	ECOG PS	LDH-ULN	WBC-10 × ^9^/L
0	< 50	0–1	< 0.67	< 6.700
1	50–59		0.67–0.99	6.700–9.999
2	60–69	2–4	1.0–1.49	10.000–14.999
3	≥ 70		≥ 1.5	≥ 15.000

Mantle cell risk classification	
MIPI score	Risk group
0–3	Low risk
4–5	Intermediate risk
> 5–11	High risk

*ECOG PS* Eastern Cooperative Oncology Group performance status, *LDH-ULN* lactic acid dehydrogenase institutional upper limit of normal, *WBC* white blood cell count

### CNS: International Prognostic Index (CNS-IPI)

The CNS—international prognostic index (CNS-IPI; Table 14) is a robust, highly reproducible tool that can be used to estimate the risk of CNS disease in patients with DLBCL [23].

**Table:** **Table 14** CNS—international prognostic index (CNS-IPI)

**Score**	**0**	**1**
Age (years)	< 60	≥ 60
Performance Status	0–1	2–4
Stage	I–II	III–IV
Lactate dehydrogenase	Normal level	≥ Normal levels
Extra-nodal sites	≤ 1	> 1
Kidneys and/or adrenal glands	No	Yes
**CNS-IPI risk group**	**Score**	**Risk (%)**
Low-risk	0–1	0.6
Intermediate risk	2–3	3.4
High risk	4–6	10.2

## Management of Lymphoma Subtypes

### Hodgkin’s Lymphoma

Early stage HL has a cure rate of 90% and hence, the risk adapted combined modality treatment is the current standard of care [24–26]. The PET scans have an active role to play in reducing treatment for early and advanced stage disease [24–26]. The 5-year survival for advanced stage disease with combined modality treatment is around 60 to 80% [27–30]. Table 15 shows the optimal management strategy for HL.

**Table:** **Table 15** Management of Hodgkin’s lymphoma [24–36]

Clinical stage	Treatment regimen
**Clinical stages I and II**	
**All histologies** **Favorable risk**	• Clinical stage 1A NLPHL—consider IFRT alone • ABVD × 2 cycles → IFRT (20 Gy) Avoid RT (especially in patients aged < 55 years with disease in mediastinum or abdomen) • ABVD × 2 → PET/CT - If PET negative, then further ABVD × 2 - If PET positive, then further ABVD × 2 followed by IFRT
Unfavorable risk* **Non-bulky** [any unfavorable risk factor*]	• ABVD × 4 cycles → IFRT [30 Gy] • ABVD × 6 cycles for patients with B symptoms or extra-nodal extension • Consider escalated BEACOPP (2 cycles) in case of PR on PET
**Bulky** • 10 cms • 1/3 maximal transthoracic diameter on X-ray	• ABVD × 6 cycles → IFRT [30 Gy] to prior bulk site • If end of treatment PET is negative RT can be avoided • Consider escalated BEACOPP (2 cycles) in case of PR on PET
**Clinical stages III and IV**	
All histologies Non bulky	• ABVD × 6 cycles • IFRT [30 Gy] if there is PET positive residual mass • Consider escalated BEACOPP (4 cycles) in case of PR on PET scan after cycle 2 of ABVD • Consider Omitting Bleomycin, if required, if in CR on PET scan after cycle 2 • *Brentuximab Vedotin based regimen (A*-*AVD) has been recently found superior to ABVD. Cost of therapy and drug import have to be considered before discussing this regimen* *Elderly patients* > 65 *years may be treated with COPP*

Bulky disease = MTD [maximal transthoracic diameter] = mediastinal mass width/maximal intrathoracic width > 1/3, or any mass 10 cms

ABVD: perform pulmonary function tests (PFT) at baseline, and after cycles 3 and 5: omit bleomycin if > 25% decrease in PFT

Bleomycin omission: from ABVD regimen after negative interim PET/CT results in lower incidence of pulmonary toxicity but not significant lower efficacy [25]

*ABVD* adriyamycin, bleomycin, vinblastine, dacarbazine, *BEACOPP* bleomycin, etoposide, adriamycin, cyclophosphamide, vincristine, procarbazine, prednisone, *COPP* cyclophosphamide, oncovin, procarbazine and prednisone, *IFRT* involved field radiotherapy, 20–30 Gy, *PET-CT* positron emission tomography computed tomography, *RT* radiotherapy, *NLPHL* nodular lymphocytic predominant Hodgkin’s lymphoma

**Table:** 

***Note:*** ***Risk Factors:*** Favourable: Stage I-II without risk factors Unfavourable*: Stage I-II with risk factors
Bulky Mediastinal mass
Age > 50 years
ESR-30 mm/1st hour if no B symptoms, and 50 mm/1st hour in presence of B symptoms
B symptoms
More than 3 nodal sites

### Non-Hodgkin’s Lymphoma: B-Cell Indolent

The optimal management strategies for low-grade NHL (i.e. FL, marginal zone lymphomas [MZL], mucosa-associated lymphoid tissue lymphoma [MALT], and chronic lymphocytic leukemia [CLL]/SLL) are described below (Tables 16, 17, 18).

**Table:** **Table 16** Management of follicular lymphoma [37–43]

Clinical stage	Treatment regimen
**Early stage**: IA or contiguous IIA	• IFRT 24 Gy 12# to 30 Gy 20# (*watchful waiting is acceptable*)
**Advanced stage**: III, IV	**No symptoms** • Watchful waiting • (*Rituximab monotherapy* × *4 weekly, *± *maintenance R* × *q3 monthly for 1* *year)*
**Indications for treatment in advanced stage** • Symptoms (fever, night sweats, weight loss, malaise, pain etc.) • Significant adenopathy: > 7 cms, ≥ 3 sites and ≥ 3 cms, rapidly progressive • Splenomegaly > 5 cms below the costal margin • Impending organ compromise (compression, pleural effusion, pericardial effusion, ascites) • Cytopenias secondary to marrow infiltration • Patient preference: anxiety and poor QoL	**Symptomatic** **Grade 1,2,3a FL** • SA Rituximab × 4 weekly followed by maintenance R × q3 monthly for 1 year • B-R × 6 • CVP-R × 6 → (± *Maintenance R* × *q3 monthly for 2* *years)* **Grade 3 a and 3 b FL** • CHOP-R × 6 → (± *Maintenance R* × *q3 monthly for 2* *years)* **Serious Co-morbidities** • Chlorambucil oral ± Rituximab or prednisolone

*B-R* bendamustine-rituximab, *CHOP-R* cyclophosphamide, doxorubicin (hydroxydaunomycin), vincristine, prednisone and rituximab, *CVP-R* cyclophosphamide, vincristine, prednisolone and rituximab, *IFRT* involved field radiotherapy, *QoL* quality of life, *R* rituximab, *SA* single agent

**Table:** **Table 17** Management of indolent lymphomas (other than follicular lymphoma)

Clinical stage	Treatment regimen
Stages 1 and 2	• Asymptomatic patients can be observed • Treat with IFRT • Combined modality chemo-immunotherapy × 3 cycles (chlorambucil, CVP, or bendamustine) → local RT
Stages 3 and 4: asymptomatic	• Observation alone • SA rituximab weekly × 4 followed by maintenance 2 to 3 monthly for 1 years
Stages 3 and 4: symptomatic	Chemo-immunotherapy × 6 cycles followed by ± maintenance rituximab for 2 years. • CVP ± R • CHOP ± R • B ± R

*B-R* bendamustine-rituximab, *CHOP-R* cyclophosphamide, doxorubicin (hydroxydaunomycin), vincristine, prednisone and rituximab, *CVP-R* cyclophosphamide, vincristine, prednisolone and rituximab, *IFRT* involved field radiotherapy, *RT* radiotherapy, *SA* single agent, *R* rituximab

**Table:** **Table 18** Management of chronic lymphocytic leukemia (CLL)/small lymphocytic lymphoma (SLL) [44–48]

Clinical stage	Treatment regimen
**Early stage** ***Rai 0***: lymphocytosis only ***Binet A***: < 3 areas of lymphadenopathy No anemia or thrombocytopenia	No treatment indicated generally^a^ Watchful waiting
**Intermediate stage** ***Rai I-II***: lymphadenopathy, splenomegaly ± hepatomegaly ***Binet B***: > 3 cms of lymphadenopathy, no anemia or thrombocytopenia	Possibly^a^
**Advanced stage** ***Rai III-IV***: Anemia, thrombocytopenia ***Binet C***: Hemoglobin < 10 gm/dL; platelet < 100 × 10^9^/L	**Always** **Fit for treatment** ***No mutation of del (17p):*** FCR × 6 (or, B-R × 6 is an option) ***Mutation and/or del (17p)***: Ibrutinib OR High dose methylprednisolone-R *In the young, due consideration for Allogeneic HSCT must be given* **Unfit for treatment with full dose FCR** ***No mutation or del(17p):*** B ± R × 6, FC-R × 6 (dose reduced), CVP ± R × 6, Chlorambucil ± R ***Mutation del (17p):*** consider ibrutinib

Absolute lymphocyte count alone is not an indication for treatment unless above 200–300 × 10^9^/L or symptoms related to leukostasis

*B-R* bendamustine-rituximab, *CVP-R* cyclophosphamide, vincristine, prednisolone and rituximab, *FC-R* fludarabine, cyclophosphamide, and rituximab, *R* rituximab, *HSCT* hematopoeitic stem cell transplant

^a^Treatment indicated when lymphocyte doubling time (LDT) is < 12 months, high LDH and β-2 microglobulin levels, massive splenomegaly > 5cms below the costal margin, or constitutional B symptoms

### Non-Hodgkin’s Lymphoma: B-Cell High Grade

The optimal management strategies for adult B-cell high grade NHL (i.e. DLBCL, mantle cell lymphoma [MCL], BL, and LBL) are given below.

#### Management of Diffuse Large B Cell Lymphoma

The treatment options vary between patients with localized (stage I-II) and advanced (stage III-IV) disease (Table 19). Prognosis is extremely good for patients with no adverse risk factors (normal LDH, stage I or II non-bulky disease, age < 60 years or ECOG performance status < 2). Five-year survival for advanced stage varies from 30 to 50%.

**Table:** **Table 19** Management of diffuse large B cell lymphoma [49–55]

Clinical stage	Treatment regimen
**Limited stage** I–II, no B symptoms, non-bulky (≤ 10 cms)	
Low IPI [0,1,2] If <***55*** ***years and wish to avoid RT*** to chest and abdomen	CHOP-R × 3 cycles → IFRT 30 Gy/15 # or 36 Gy/20# CHOP-R × 4 for IPI-0 CHOP-R × 6 for IPI – 1 or 2
High IPI [3, 4]	CHOP-R × 6 + IFRT 30–36 Gy
**Advanced stage** III–IV, B symptoms, bulk ≥ 10 cms	
Low IPI [1,2] and/or Age > 65 years	CHOP-R × 6 ± RT CEOP-R × 6 ± RT or mini CHOP-R × 6
High IPI [3,4,5] Young patient with Mediastinal Large B-cell Lymphoma, intermediate between DLBCL and Burkitt’s or Double Hit [DH] lymphoma	CHOP-R × 6 ± RT da EPOCH—R × 6 cycles

Patients with bulky disease or impaired renal function should be monitored for tumor lysis syndrome. Doxorubicin in CHOP regimen can be replaced with etoposide (CEOP), liposomal doxorubicin or mitoxantrone in patients with poor left ventricular function (Category 2B); elderly patients above the age of 80 years may receive mini CHOP-R

PET/CT scan at interim restaging can lead to increased false positives and should be carefully considered in select cases. If PET/CT scan performed and positive, rebiopsy before changing course of treatment

*CHOP-R* cyclophosphamide, doxorubicin (hydroxydaunomycin), vincristine, prednisone and rituximab, *DA-EPOCH-R* dose adjusted etoposide, prednisone, oncovin (vincristine), cyclophosphamide, hydroxydaunorubicin (doxorubicin), and rituximab; *DLBCL* diffuse large B cell lymphoma, *IFRT* involved field radiotherapy, *IPI* international prognostic index; *mini CHOP-R* rituximab combined with low-dose CHOP, *RT* radiotherapy, *R* rituximab

In selected cases, RT to bulky sites may be beneficial (Category 2B). Patients at increased risk of CNS relapse (those with high CNS-IPI, involvement of the paranasal sinuses, testes, breast, bone-marrow involvement with large cells or having ≥ 2 extra-nodal sites with elevated LDH, mediastinal large B cell lymphoma and DHL) must undergo CSF cytology and should receive CNS prophylaxis with 4–8 doses of intrathecal methotrexate. An alternative is to consider 3–3.5 g/m^2^ of high dose methotrexate during treatment. Patients with CNS involvement or CSF involvement should be considered for CNS directed therapy with 3–3.5 g/m^2^ of systemic methotrexate on day 15 of CHOP-R cycles 1, 3 and 5. Elderly patients may be given 1.0 g/m^2^ after completing their systemic treatment (*data to support and contrary available*).

#### Management of Mantle Cell Lymphoma

The treatment options for mantle cell lymphoma are given in Table 20.

**Table:** **Table 20** Management of mantle cell lymphoma [56–59]

Clinical stage	Treatment regimen
**Early Stage** Stages 1–II	IFRT (30–36 Gy) alone CHOP ± R × 6
**Advanced Stage**	
Stages II (bulky)	CHOP ± R × 6 cycles maintenance R q 2–3 monthly for 2 years
Stages III–IV [Asymptomatic patient with Low Ki—67 and low IPI]	Watchful waiting
Stages III–IV [symptomatic patient]	**Fit for auto HSCT** CHOP-R alternate with DHAP-R × 6 → HDT and auto HSCT in remission^a^→maintenance Rituximab **Unfit for auto HSCT** B-R × 6 cycles ± maintenance R q 2–3 monthly for 2 years CHOP-R × 6 cycles ± maintenance R q 2–3 monthly for 2 years CVP ± R × 6 ± maintenance R Chlorambucil ± R

For patients not achieving at least PR with first line therapy, second line therapy may be considered in an effort to improve the quality of a response before they are taken for consolidation with HDT and Auto HSCT

*CHOP-R* cyclophosphamide, doxorubicin (hydroxydaunomycin), vincristine, prednisone and rituximab, *HD* high dose, *IFRT* involved field radiotherapy, *IPI* international prognostic index, *mini CHOP-R* rituximab combined with low-dose CHOP, *DHAP-R* dexamethasone, high dose Ara-C cytarabine, platinol (cisplatin) and rituximab, *HSCT* hemopoeitic stem cell transplantation, *RT* radiotherapy, *R* rituximab

^a^For young patients with CR or PR to first line therapy, consolidation with high dose therapy (HDT) autologous hematopoietic stem cell transplant (Auto HSCT) is recommended

Less aggressive therapies like B-R are recommended for elderly patients, cardiac compromise and patients unfit to tolerate aggressive regimens. Maintenance rituximab is recommended for patients who are not candidates for high dose therapy autologous hematopeitic stem cell transplant (HDT/auto HSCT) and are in remission after first line therapy with R-CHOP.

#### Management of Burkitt’s Lymphoma (BL)

There is a high incidence of tumor-lysis syndrome and measures should be taken to prevent and treat this complication. Patients with bulky disease and organ dysfunction may be treated with modified dose therapy (e.g. pre-phase-CVP), in an attempt to modify the effects of tumor lysis. Then, a more intensive therapy needs to be administered as outlined below [60, 61].

- Dose adjusted etoposide, prednisone, oncovin (vincristine), cyclophosphamide, hy-droxydaunorubicin (doxorubicin) ± rituximab (daEPOCH ± R)
- Berlin-Frankfurt-Münster (BFM) protocol (B-NHL 2002)
- Hyperfractionated cyclophosphamide, vincristine, doxorubicin (Adriamycin), and dexamethasone ± rituximab (Hyper CVAD ± R)

#### Management of Lymphoblastic Lymphoma (LL)

Patients with LL are typically managed (including diagnostics) and treated with regimens appropriate for acute lymphoblastic leukemia (ALL). Patients with systemic LL can be treated with any one of the chemotherapy regimens:

- MCP-841 protocol
- German multicenter ALL (GMALL) protocol
- Hyper-CVAD alternating with high dose methotrexate and cytarabine

Young adults may be considered for pediatric based ALL protocols, based on center experience. Patients with complete response (CR) to induction therapy should be continued with other components of the treatment protocols. It is important that patients be treated with a given treatment protocol in its entirety and not be treated with different components taken from different protocols. Patients with high risk features (such as marrow involvement) and with a matched sibling donor should be offered an allogeneic transplantation in first remission.

### Non-Hodgkin’s Lymphoma: T Cell Lymphoma [62–74]

The T-cell malignancies are rare and often complex diseases. Diagnosis and management should be discussed in a multi-disciplinary team meeting and those patients requiring treatment should generally be referred to a cancer centre or tertiary centre with specialist expertise. The rarity of these diseases and the lack of randomized trials mean that there is no consensus about optimal therapy for T- and NK-cell neoplasms and recommendations are therefore based on small case series, phase II trials and expert opinion.

#### Nodal Peripheral T-Cell Lymphoma

##### Peripheral T Cell Lymphoma Not Otherwise Specified (PTCLnos)

Treatment with an anthracycline-based chemotherapy regimen—6 cycles of CHOP (or CHOEP) is recommended. The option of autologous HSCT as a consolidative measure may be considered in patients eligible for transplant, having achieved or having an ongoing response, and in those with high risk disease.

##### Anaplastic Large Cell Lymphoma (ALCL)

*Limited stage:* ALK-positive ALCL and no adverse prognostic features by IPI should be treated with 3–4 cycles of CHOP chemotherapy and IFRT. A younger fit patient (adolescent young adults) may be considered for the more intensive short course BFM protocol for NHL which includes high dose methotrexate.

*Advanced stage:* Patients should receive 6–8 cycles of CHOP chemotherapy.

In ALK-negative ALCL, consider checking DUSP22 gene rearrangement. ALK negative DUSP22 positive ALCL can be treated similar to ALK-positive ALCL [75].

ALK-negative ALCL should be treated as for PTCL-NOS (peripheral T-cell lymphoma not otherwise specified). A younger fit patient (adolescent young adults) may be considered for the more intensive short course BFM protocol for NHL which includes high dose methotrexate. CHOEP is an alternative regimen, for ALK-negative advanced stage lymphoma (however, there is insufficient data to recommend). Consideration should be given to consolidation with auto-HSCT.

##### Angioimmunoblastic T Cell Lymphoma (AITL)

Treatment with CHOP (or CHOEP) is recommended followed by consolidation with HD chemotherapy and auto HSCT. The use of GDP protocol as an alternate to CHOP may be considered from the toxicity perspective with equivalent results. In patients with a relative indolent disease the option of using cyclosporine for inducing response may be considered in relapses following primary therapy.

#### Mature T-Cell Leukemia

##### T-prolymphocytic leukemia (T-PLL):

Single agent pentostatin 4 mg/m^2^ every week × 4 → x 2 weekly till maximum response.

Alternative regimens include fludarabine, cyclophosphamide, mitoxantrone (FCM) combination, and the use should be considered with individual center experience and access to the drugs. Alemtuzumab, a drug commonly used in this condition is not currently available in India, and can potentially be imported.

##### T- large granular lymphocytic leukemia (T-LGL):

 The management of T-LGL is provided in Table 21.

**Table:** **Table 21** Management of T-large granular lymphocytic leukemia (LGL)

T-LGL presentation	Treatment regimen
Asymptomatic	Watchful waiting
Mild cytopenia—Hemoglobin < 9 gm/dL	Packed red blood cell transfusions
Severe cytopenia—ANC < 500/mm^3^ Platelets < 50,000/mm^3^	Methotrexate (MTX) is preferred as a first line and CTX is considered in case of MTX failure • MTX SA 10 mg/m^2^/week or • Cyclophasphamide 50 to 100 mg/day as single agent or • Cyclosporin 5 to 6 mg/kg/day in 2 divided doses (considered in case of failure to both MTX and CTX) or • Fludarabine/cladirabine/bendamustine or • Splenectomy in select patients

*CTX* cyclophosphamide, *MTX* methotrexate, *SA* single agent

##### Chronic lymphoproliferative disease of NK cells (CLPD-NK):

 Management as for T-LGL.

##### Aggressive NK cell leukemia:

 Younger patients must be treated with ALL based protocols.

##### Adult T cell leukemia lymphoma (ATLL):

The management of ATLL is provided in Table 22.

**Table:** **Table 22** Management of ATLL

ATLL presentation	Treatment regimen
Smouldering	No benefit from early treatment—wait and watch
Lymphoma	CHOP + concurrent AZT followed by allogeneic HSCT in first remission
Leukemia/high-grade [HG] lymphoma	CHOP + concurrent AZT followed by allogeneic HSCT in first remission CNS prophylaxis as for HG DLBCLs

*AZT* zidovudine, *CHOP-R* cyclophosphamide, doxorubicin (hydroxydaunomycin), vincristine, and prednisone, *CNS* central nervous system, *DLBCL* diffuse large B-cell lymphoma, *HG* high grade, *HSCT* hemopoeitic stem cell transplantation

#### Extranodal Peripheral T-Cell Lymphomas

##### Cutaneous T-Cell Lymphomas (CTCL)

The CTCL may present with a chronic, patchy infiltrative skin disorder (mycosis fungoides—50% of cutaneous lymphomas) or with a diffuse erythema and malignant T-cells in the peripheral blood (Sezary syndrome) (Table 23).

**Table:** **Table 23** Management of cutaneous T cell lymphomas

Clinical stage	Treatment regimens
Stages I–II A	Topical corticosteroids, nitrogen mustard ointment
Failure of topical treatment	Psoralen and ultra violet A radiation (PUVA)
Stages III–IV	Total skin electron beam therapy (TSET) ***Systemic therapies*** Single agent methotrexate (≤ 100 mg/week) Chlorumbucil Cyclophosphamide Retinoids Interferon Brentuximab vedotin (in CD30 +) ***Combination therapies*** CHOP Fludarabine/Cladarabine ± Mitoxantronebased (FC/FCM) Gemcitabine based (GDP)

*CHOP* cyclophosphamide, doxorubicin (hydroxydaunomycin), vincristine, and prednisone, *FCM* fludarabine, cyclophosphamide and mitoxantrone, *GDP* gemcitabine, dexamethasone, and cisplatin

##### Extranodal NK/T Cell Type Lymphoma, Nasal Type

- *Stages 1 and II:* modified SMILE × 4 cycles followed by local RT is recommended. RT (55 Gy) as a single modality is recommended for smaller lesions
- *Advanced stage* disease (III and IV): modified SMILE × 6 cycles followed by local RT is recommended.

*Enteropathy associated T cell lymphoma (EATL)*: CHOP like therapy ± autograft in first remission.

*Hepatosplenic T cell lymphoma*: No satisfactory recommendations. Treatment as applied for PTCL-NoS with CHOEP × 3 to 4 cycles followed by consideration for HDT and autologous transplant.

*Subcutaneous panniculitis T*-*cell lymphoma*: No recommendations per se; however, cyclosporine-A can be considered, especially in the presence of α/β type with CD8 positive and CD 56 negative entities. CHOP like chemotherapy may be considered in case of failure of cyclosporine A. Single agent methotrexate has been found useful in some patients.

## Special Issues in Lymphoma Management

### HIV-Associated Lymphoma

Treatment options for HIV-associated Burkitt’s lymphoma include daEPOCH, CODOX-M/IVAC, or hyper-CVAD ± R. DLBCL should be treated with short course (sc) EPOCH ± R or CHOP ± R. Most cases of primary effusion lymphoma (PEL) are CD20-negative; the addition of rituximab to CHOP is not indicated. Plasmablastic lymphoma (PBL) can be treated with regimens recommended for Burkitt’s lymphoma. High-dose methotrexate or RT can be considered for patients with primary CNS lymphoma (PCNSL) as suggested below.

Early introduction of highly active antiretroviral therapy (HAART) is associated with superior outcomes. Patient should receive HAART and growth factor support along with full-dose chemotherapy. In patients with persistently low CD4 counts (< 100/µL), rituximab should be omitted to reduce the risk of serious infections.

### Primary CNS Lymphoma and Primary Intra-ocular Lymphoma

**Fig. 1:**
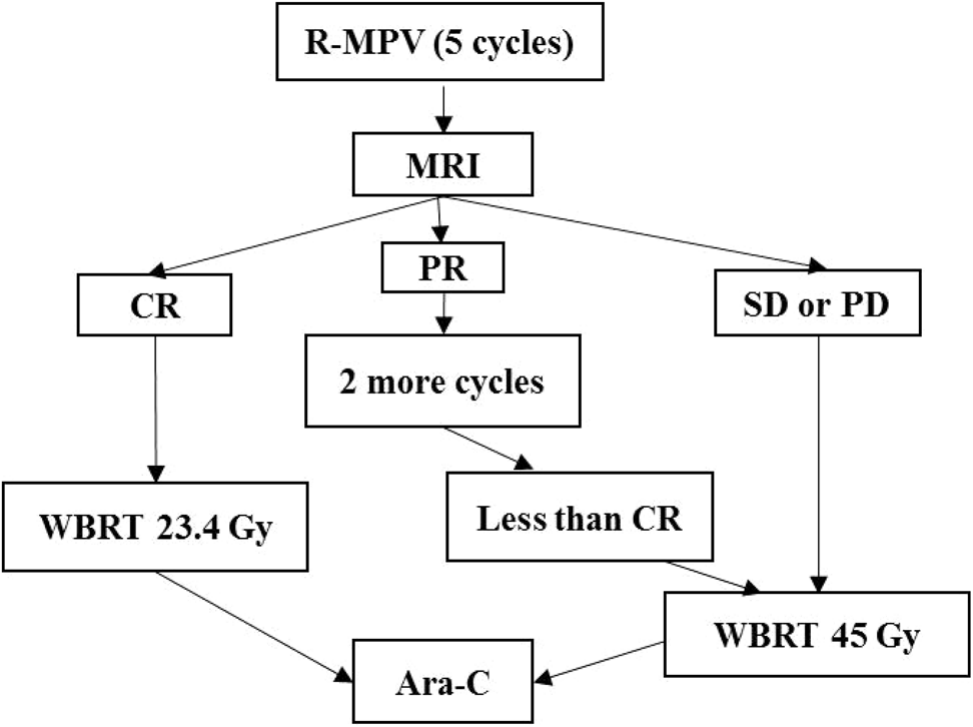
Treatment algorithm of Primary CNS lymphoma (PCNSL). *Ara-c* cytarabine, *CR* complete response, *MRI* magnetic resonance imaging, *PD* progressive disease, *PR* partial response, *R-MPV* rituximab, methotrexate, procarbazine, and vincristine, *SD* stable disease, *WBRT* whole brain radiation therapy

Chemotherapy should consist of a regimen that includes high-dose methotrexate (if the histology is DLBCL/BL) (Fig. 1).

- MVP-R × 5–7 cycles
- Consolidation WBRT, 45 Gy in 25 fractions (or 23.4 Gy), should be considered in patients who achieve CR with MTX-based chemotherapy; followed by 2 doses of HD cytosine arabinoside × 2 cycles
- Alternative regimens include whole brain radiation therapy (WBRT) along with temozolomide ± methotrexate
- Institutions with adequate expertise can consider the options of intensive therapies like MATRix protocol or a high dose chemotherapy and autologous stem cell rescue consolidation approach [76].

There is no role for CHOP-like chemotherapy in the treatment of primary CNS lymphoma (PCNSL).

In patients under 60 years of age, WBRT should be offered to patients unless there is a significant neurocognitive deficit following chemotherapy. In patients aged 60 years or over, neurocognitive side-effects are more likely to outweigh potential benefits.

### Primary Testicular Lymphoma (PTL)

Patients with limited disease should be managed with primary orchidectomy followed by CHOP-R treatment, CNS prophylaxis (intrathecal chemotherapy ± high-dose methotrexate or high-dose cytarabine) and prophylactic scrotal radiotherapy.

*Stage IE*: CHOP-R × 6 cycles followed by scrotal RT (25–30 Gy), including RT to the contralateral testis), along with four doses of intrathecal methotrexate (starting from day 1 of CHOP-R).

**Fig. 2:**
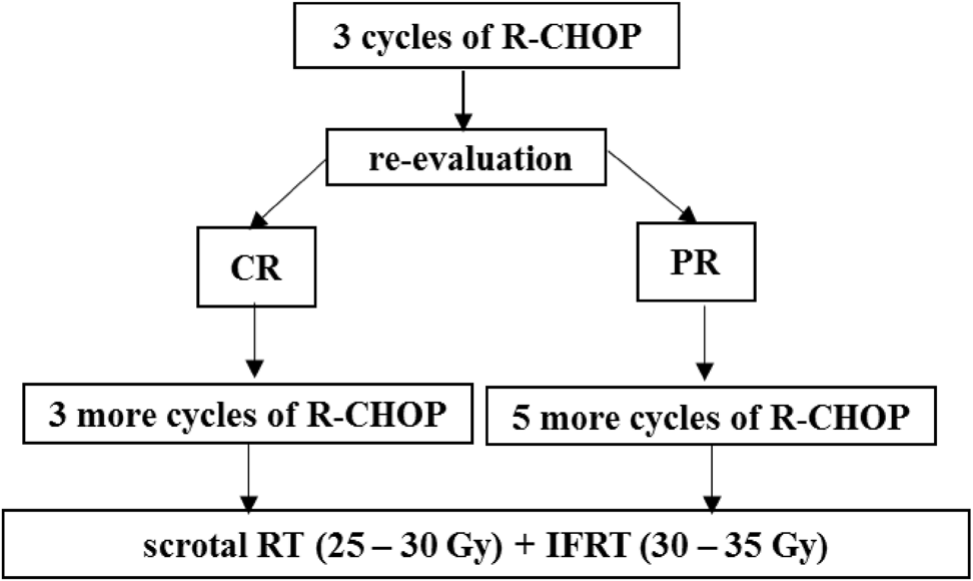
Treatment for Stage II E Primary Testicular Lymphoma (PTL). Four doses of intrathecal methotrexate (starting on day 1 of cycle I R-CHOP)

*Stage IIE*: Fig. 2 represents the treatment for stage II E disease.

*Management of Advanced Stage Disease (Stage III–IV)*: Should be treated according to the guidelines for the treatment of advanced stage DLBCL with CHOP-R × 6 to 8 cycles along with prophylactic scrotal radiotherapy and intrathecal chemotherapy.

The addition of intermediate-high dose methotrexate might improve CNS prophylaxis, especially in the younger patients but this has never been formally demonstrated. High-dose chemotherapy followed by stem cell transplantation is an investigational option.

### Primary Gastrointestinal Lymphoma

Treatment is according to histological subtype. Resection of gastrointestinal lymphoma is no longer recommended, unless necessary to establish a definite diagnosis or to control the complications of hemorrhage or perforation.

### Primary Cutaneous B-Cell Lymphoma (CBCL)

In the WHO-European Organization for Research and Treatment of Cancer (EORTC) classification, three main types of CBCL are distinguished, which are primary cutaneous marginal zone lymphoma (PCMZL), primary cutaneous follicle center lymphoma (PCFCL) and primary cutaneous diffuse large B cell lymphoma, leg type (PCLBCL-LT). The PCMZL and PCFCL are indolent types and PCLBCL-LT has an unfavorable outcome (Table 24) [2].

**Table:** **Table 24** Management of primary cutaneous B cell lymphoma

**PCMZL/PCFCL**	**First-line**	**Alternative**
Solitary/Localized	Local radiotherapy, Excision, Wait and Watch	Intralesional steroids, Topical steroids, Intralesional Rituximab
Multifocal	Local radiotherapy, Chlorambucil	Rituximab SA, CVP-R
**PCLBCL, LT**		
Solitary/Localized	CHOP-R ± IFRT	
Multifocal	CHOP-R	

*CHOP-R* cyclophosphamide, doxorubicin (hydroxydaunomycin), vincristine, prednisone and rituximab; CVP-R cyclophosphamide, vincristine, prednisolone and rituximab, *IFRT* involved field radiotherapy, *PCFCL* primary cutaneous follicle center lymphoma, *PCLBCL LT* primary cutaneous diffuse large B cell lymphoma, leg type, *PCMZL* primary cutaneous marginal zone lymphoma, *SA* single agent

## Management of Relapsed Lymphoma

### Pretreatment Evaluation

1. Histopathological examination with a basic immune-histochemistry diagnostic algorithm is mandatory in the evaluation of relapsed disease. Additional molecular investigations are desirable and will be based on the institutional practice.
2. In the relapsed indolent lymphomas, always rule out Richter’s Transformation
3. Subtype specific prognostication of the disease status is highly recommended
4. Re-staging as appropriate to disease subtype is mandatory, and would include whole body PET-CT (or institutional practice) and bone marrow biopsy.
5. Infectious disease screening is required to rule out blood borne viral diseases (e.g., HBsAg, HCV, HIV)
6. Co-morbidity assessment for co-existing medical conditions and fitness for intensive therapy (like HCT) is mandatory (liver and renal function tests, echocardiography/multigated acquisition scan [MUGA] scan, etc.). Assigning a co-morbidity score is desirable.

### Management Approach for Relapsed Lymphoma

**Fig. 3:**
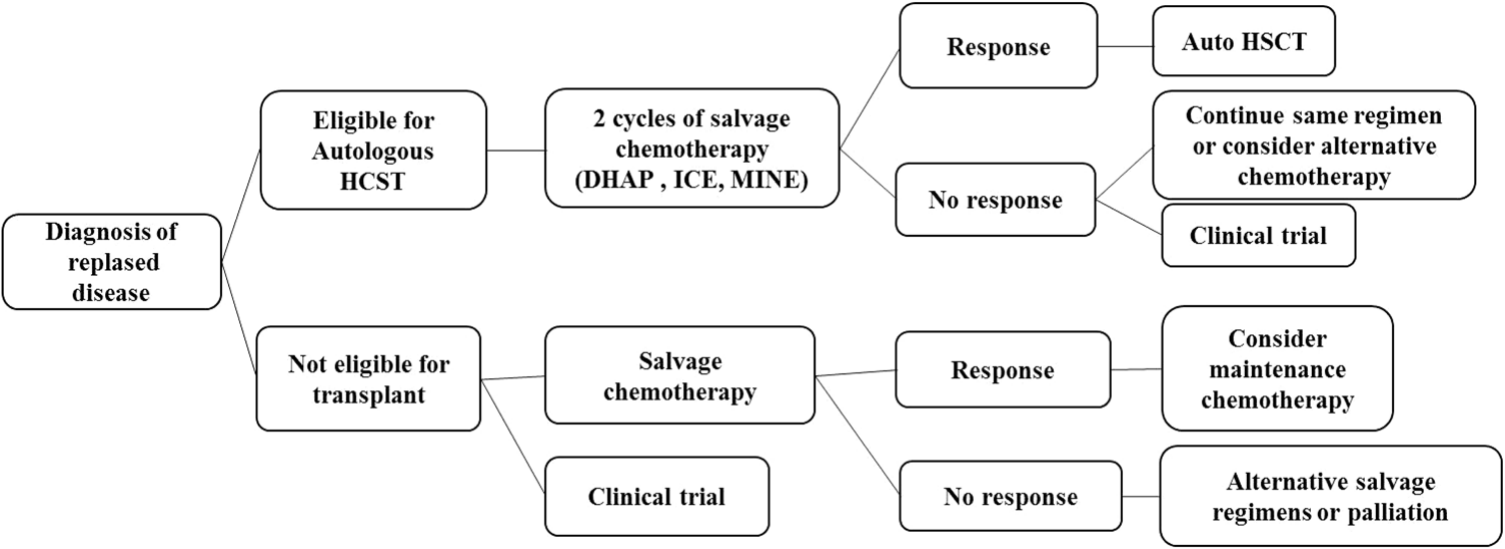
Management algorithm for relapsed lymphoma. *HSCT* hematopoietic stem cell transplant, *DHAP* dexamethasone, high dose cytosine arabinoside, cisplatin, *ICE* ifosphamide, carboplatin, etoposide, *MINE* mesna, ifosphamide, mitroxantrone, etoposide

A suggested management algorithm for relapsed lymphoma is shown in Fig. 3.

### Chemotherapy Regimens for Transplant Eligible Patients [77–85]

Selection of second-line chemotherapy regimens depends on the pattern of relapse and the agents previously used. Platinum compound based regimens have been associated with good responses and lower levels of myelotoxicity and are widely used for salvage chemotherapy in potential transplant candidates. These include:

- DHAP (dexamethasone, cisplatin, cytarabine) ± rituximab
- ICE (ifosfamide, carboplatin, etoposide) ± rituximab
- ESHAP (etoposide, methylprednisolone, cytarabine, cisplatin) ± rituximab
- GDP (gemcitabine, dexamethasone, cisplatin) ± rituximab [carboplatin substitution for cisplatin is an acceptable alternative]
- GemOx (gemcitabine, oxaliplatin) ± rituximab
- MINE (mesna, ifosfamide, mitoxantrone, etoposide) ± rituximab

**Table:** 

*Note:* 1. Use of additional anthracyclines must be accompanied by careful monitoring of the cardiac status. 2. Disease status should be evaluated with imaging studies and clinical assessment after two to three cycles, following which autologous HSCT should be carried out.	

### High-Dose Chemotherapy Regimens Commonly Used in Autologous HSCT

- BCNU, cyclophosphamide, cytosine arabinoside and melphalan (BEAM) ± rituximab
- Busulfan and cyclophosphamide (Bu-Cy) ± rituximab
- Melphalan, busulfan, and total body irradiation (TBI) ± rituximab
- Cyclophosphamide (with or without etoposide) plus TBI ± rituximab
- Bendamustine, etoposide, cytarabine, melphalan (BeEAM) ± rituximab
- Thiotepa, busulfan, and cyclophosphamide (TBC) ± rituximab
- Lomustine (CCNU), cytarabine (Ara-C), cyclophosphamide, etoposide (LACE) ± rituximab

Role of double/tandem transplant is still experimental and evidence is not mature.

### Role of Allogeneic HSCT

Allogeneic HCT may be considered in young patients who are considered fit to undergo intensive conditioning therapies, and have any one of the following,

- Stem cell mobilization failure, or
- Relapse after autologous HCT, or
- High risk/aggressive disease: upfront use in select patients (< 40 years). These decisions must be made after a multidisciplinary consensus (e.g. primary refractory disease in the young responding to salvage chemotherapy, bone marrow involvement post induction chemotherapy, etc.)

Alternative donor sources, reduced intensity conditioning, etc. are still experimental and no guidelines exist for the same.

### Salvage Chemotherapy in Transplant Ineligible Patients [85–92]

- Participation in clinical trials with new agents highly recommended whenever available
- Frail individuals
  - CEPP (cyclophosphamide, etoposide, prednisone, procarbazine) ± rituximab
  - Lenalidomide ± rituximab
- Patients with ECOG performance status > 2
  - da-EPOCH ± rituximab
  - GDP ± rituximab
  - GemOx ± rituximab

## Newer Therapy Options

### Indolent B-Cell Lymphoma

*Chronic Lymphatic Leukemia/Small Lymphocytic Leukemia*

Consider participation in clinical trial with new agents.

1. Ibrutinib
2. Venetoclax (post-ibrutinib)
3. Idelalisib
4. Obinutuzumab or ofatumumab (especially in rituximab refractory)
5. Chemo-immunotherapy
  1. Rituximab (or obinutuzumab in rituximab refractory)
  2. Chemotherapy: fludarabine-cyclophosphamide v/s. CHOP v/s. ibrutinib-bendamustine, etc.
6. Non-chemo combination therapies
  1. Ibrutinib + Venetoclax
  2. Rituximab + Ibrutinib
  3. Rituximab + Venetoclax
7. Post-induction maintenance therapy must be considered in patients who have partial or complete response.
8. p53 mutated (or 17p deleted) disease is generally resistant to conventional therapies. In this subset of patients, allogeneic bone marrow transplant (BMT) must be considered in the young (especially those with a complex karyotype).

*Follicular Lymphoma*

Consider clinical trial recruitment.

1. Alternative chemo-immunotherapy not used upfront (e.g. B-R v/s R-CVP v/s R-CHOP)
2. Obinutuzumab (in riuximab refractory)
3. Idelalisib (rituximab and chemotherapy refractory)
4. Post-induction maintenance therapy must be considered in patients who have partial or complete response.

### Hodgkin’s Lymphoma

*First-line Salvage Therapy*

1. In very selected patients with favorable risk localized late relapse: local RT alone may suffice
2. High dose Chemotherapy regimens, as recommended
  Other regimens to be considered: mini-BEAM
3. In refractory disease setting, patients who are salvage chemotherapy responsive: consider post-transplant maintenance therapy with brentuximab vedotin (BV) for 1 year.
4. Role of consolidation radiation therapy must be made in the light of the site(s) of relapse, rapidity of relapse, response to salvage therapy and prior radiotherapy. There is limited evidence regarding the timing of radiotherapy and transplant.

*Subsequent Salvage Therapy*

1. Consider recruitment in clinical trials
2. Brentuximab vedotin (or combination therapies with BV)
3. In BV exposed patients: consider PDL1 checkpoint blockade therapy with nivolumab or pembrolizumab.
4. In fit patients, consolidate with an allogeneic HCT.
5. Alternative options
  Non-cross resistant combination chemotherapy
  Single agent therapy: bendamustine v/s. everolimus v/s. lenalidomide
6. Role of directed or consolidation radiation therapy must be made in the light of the site(s) of relapse, rapidity of relapse, response to salvage therapy and prior radiotherapy.
7. There is limited evidence regarding the timing of radiotherapy and transplant.

### Aggressive or High-Grade B-Cell Lymphoma

*Burkitt’s Lymphoma*

Limited studies and regimens available. These are not Level-I or Level-II recommendations, and consider clinical trial recruitment.

1. Alternative non-cross resistant therapy to the primary regimen used:
  e.g. R-daEPOCH v/s R-ICE v/s R-GDP
2. CNS Prophylaxis always indicated
3. In the young and selected patients: always consider allogeneic HCT consolidation instead of autologous HCT
4. Additional local radiation therapy, as appropriate

*Mantle Cell Lymphoma (Non-indolent Subtype)*

Consider Clinical trial recruitment.

1. Ibrutinib alone or in combination (e.g., ibrutinib-lenalidomide-rituximab)
2. Bortezomib–rituximab (or bendamustine-bortezomib-rituximab)
3. Cladribine–rituximab OR fludarabine-cyclophosphamide-rituximab
4. Venetoclax (post-ibrutinib)
5. In the fit patient: always consolidate with an allogeneic HCT
6. Additional local radiation therapy, as appropriate.

### T-Cell Lymphoma

*Peripheral T Cell Lymphoma (PTCL)*

1. High-dose chemotherapy regimens, as recommended
2. Other options
  ALCL (Alk positive) and CD30 positive PTCL: brentuximab vedotin
  Chemotherapy: bendamustine, pralatrexate
  Romidepsin (especially in AITL)
  Lenalidomide
  AITL: Role for cyclosporine
  Belinostat
3. Proceed to allogeneic HCT in the subset of fit patients who have a greater than partial response.
4. Additional local radiation therapy, as appropriate.

## Follow-Up of a Patient and Immunization

Patients should be followed-up every 3–4 months for the first 1 year, followed by 6 monthly for the next 2 years, and then annually. The following format is advised (Table 25).

1. Accurate history
2. Careful physical examination
3. Hematological investigation
4. Documentation of side effects: late effects of treatment
5. Documentation of relapse or second primary

**Table:** **Table 25** Follow-up interval and tests performed

Interval	Test
Every visit	• Examination of nodes, thyroid, lung, abdomen and skin • CBC with differential, LDH (+ ESR for HL) • X-ray chest annually for first 3 years in patients with intrathoracic disease
Annually	• TSH (if thyroid is irradiated) • Mammogram after age 40 years if irradiated (or after 50 years) • Influenza vaccine
Routine body scans	• After 6 weeks to 3 months of therapy • If residual disease on completion scan, CT scan/PET has to be repeated after 6 months • *Surveillance CT/PET scan has no role in the patient follow up as of date and must be used judiciously*

*CBC* complete blood count, *CT* computed tomography, *ESR* erythrocyte sedimentation rate, *HL* Hodgkin’s lymphoma, *LDH* lactate dehydrogenase, *PET* positron emission tomography, *TSH* thyroid stimulating hormone

### Immunization

The normal vaccination schedule to prevent flare of viral infections is given in Table 26.

**Table:** **Table 26** Immunization in lymphoma

Type of immunization	When should it be given?	Dose and administration
Hepatitis B vaccine	At the time of diagnosis	Hepatitis B vaccines are routinely given intramuscularly in the upper arm or anterolateral thigh For accelerated immunization schedule vaccine to be administered at 0, 1, 2, and 12 months Post vaccination immunity for Hepatitis B surface antibody has to be tested 6 weeks after completion of the immunization. HBsAb titer of > 10 miu/mL is taken as immune/hypo-responder
Influenza vaccine	Every year, in the Apr-May or Sep–Oct	0.5 mL intramuscular injection. However, individuals with a bleeding disorder should be given vaccine by deep subcutaneous injection to reduce the risk of bleeding
Pneumococcal vaccine	At the time of diagnosis, if the pneumococcal vaccine can be given at least 2 weeks before initiation of anti-lymphoid cancer treatment. If that is not possible, delay until at least 6 months after completion of all lymphoid cancer treatment and any other immunosuppressive treatment *Repeat again once* 5 *years later*	Single 0.5-mL dose administered intramuscularly or subcutaneously. Vaccines are given into the upper arm in adults **CDC recommended schedule** Conjugate vaccine 13v 0.5 ml intramuscularly or subcutaneously followed by Polysaccharide vaccine 23v 0.5 ml 8 weeks later and then Polysaccharide 23v vaccine after 5yrs
Tetanus/diphtheria	*Every* 10 *years*	0.5 mL. Vaccines are routinely given intramuscularly into the upper arm or anterolateral thigh
Meningococcal Men-ACYW vaccine	If the spleen is to be removed or to be treated with radiation, all 3 doses need to be given at least 2 weeks before splenectomy. If spleen is already removed, doses need to be given 2 weeks after splenectomy	0.5 mL given intramuscularly into the upper arm or anterolateral thigh. Two doses of MenACWY should be administered *Repeat MenACYW every* 5 *years, administered* at least 2 months apart
Hemophilus influenza type b vaccine	*Single dose*	0.5 mL given intramuscularly into the upper arm or anterolateral thigh
Polio vaccine	Oral polio vaccine ***should never be taken by*** patients with lymphoid cancer It has been replaced by inactivated polio vaccine, which is safe for patients with lymphoid cancer. IPV catch-up schedule: 2 doses 2 months apart followed by a booster after 6 months from first dose	0.5 mL given intramuscularly into the upper arm or anterolateral thigh
Measles Mumps Rubella Yellow fever BCG Intra-nasal Influenza Varicella (chicken pox) vaccine	***Never.*** (live attenuated virus) Contraindicated in immunocompromised patients	

## Appendix 1: Common Regimens [Alphabetically]

**Table:**

**ABVD**
Adriamycin (doxorubicin) 25 mg/m^2^ iv d1 and d15 Bleomycin 10 units/m^2^ iv d1 and d15 Vinblastin 6 mg/m^2^ iv d1 and d15 DTIC 375 mg/m^2^ iv d1 and d15
**B-R**
Rituximab 375 mg/m^2^ iv d1 Rituximab 500 mg/m^2^ iv d1, cycle 2–6 [for CLL] Bendamustin 90 mg/m^2^ iv on d1 and d2.
**CALGB 9111**
**Cycle 1 (4 weeks)** Cyclophosphamide 1200 mg/m^2^ iv d1 Doxorubicin (Adriamycin) 45 mg/m^2^/d iv d1, 2, 3 Vincristine 2 mg iv d1, 8, 15, 22 Prednisone 60 mg/m^2^ po or iv qd d1-21 L-Asparaginase 6000 IU/m^2^ sc or im d5, 8, 11, 15, 18, 22 ***Reduce doses if patients older than 60:*** Cyclophosphamide 800 mg/m^2^ iv d1 Doxorubicin (Adriamycin) 30 mg/m^2^/d iv d1, 2, 3 Prednisone 60 mg/m^2^ po qd d 1–7 G-CSF 5 µg/kg sc qd d4 till absolute neutrophil count (ANC) > 1000/uL **Cycle 2 (4 weeks, repeat once)** Cyclophosphamide 1000 mg/m^2^ iv d1 6-Mercaptopurine (6-MP) 60 mg/m^2^/d po d1-14 Cytarabine (Ara-C) 75 mg/m^2^/d sc d1-4 and 8–11 Vincristine 2 mg iv d15, 22 L-Asparaginase 6000 IU/m^2^ sc or im d15, 18, 22, 25 Intrathecal Methotrexate (MTX) 15 mg d1 G-CSF 5 µg/kg scqd d2 till ANC > 5000/uL **Cycle 3 (12 weeks)** 6-Mercaptopurine (6-MP) 60 mg/m^2^/d po d1-70 Methotrexate (MTX) 20 mg/m^2^ po d36, 43, 50, 57, 64 Intrathecal Methotrexate (MTX) 15 mg d1, 8, 15, 22, 29 Brain radiation 24 Gy d1-12 **Cycle 4 (8 weeks)** Doxorubicin (Adriamycin) 30 mg/m^2^/d iv d1, 8, 15 Vincristine 2 mg iv d1, 8, 15 Dexamethasone (Decadron) 10 mg/m^2^/d pod1-14 Cyclophosphamide 1000 mg/m^2^ iv d29 6-Thioguanine 60 mg/m^2^/d po d29-42 Cytarabine (Ara-C) 75 mg/m^2^/d sc d29-32 and 36–39 **Cycle 5 (16 months)** Vincristine 2 mg iv d1 Prednisone 60 mg/m^2^/d d1-5 Methotrexate (MTX) 20 mg/m^2^/d po d1, 8, 15, 22 6-Mercaptopurine (6-MP) 60 mg/m^2^/d po d1-28
**CALGB 9251**
**Cycle 1** Cyclophosphamide (Cytoxan) 200 mg/m^2^/d iv d1-5 Prednisone 60 mg/m^2^/d po d1-7 **Cycles 2, 4, 6** Ifosfamide 800 mg/m^2^/d iv over 1 h d1-5 Mesna 200 mg/m^2^ iv at 0, 4 and 8 h after ifosfamide d1-5 Methotrexate (MTX) 150 mg/m^2^ iv over 30 min d1, followed by 1350 mg/m^2^ civi over 23.5 h Leucovorin 50 mg/m^2^ iv 36 h after start of MTX, followed by 15 mg/m^2^ iv q 6 h till MTX level < 0.05 uM Vincristine 2 mg iv d1 Cytarabine (Ara-c) 150 mg/m^2^/d civi d 4 and 5 Etoposide (VP-16) 80 mg/m^2^/d iv over 1 h d 4 and 5 Dexamethasone (Decadron) 10 mg/m^2^/d po d1-5 **Cycles 3, 5, 7** Cyclophosphamide 200 mg/m^2^/d iv d1-5 Methotrexate (MTX) 150 mg/m^2^ iv over 30 min d1, followed by 1350 mg/m^2^ civi over 23.5 h Leucovorin 50 mg/m^2^ iv 36 h after start of MTX, followed by 15 mg/m^2^ iv q6 h till MTX level < 0.05 uM Vincristine 2 mg iv d1 Doxorubicin (Adriamycin) 25 mg/m^2^/d iv bolus d 4 and 5 Dexamethasone (Decadron) 10 mg/m^2^/d po d1-5 **Intrathecal (cycle 2–7)** Methotrexate (MTX) 15 mg d1 Cytarabine (Ara-c) 40 mg d1 Hydrocortisone 50 mg d1 **Brain radiation** 24 Gy post chemotherapy if bone marrow involvement Start cycle 2 right after cycle 1, cycle 2–7 are given q3w
**CEPP**
Cyclophosphamide 600 mg/m^2^ iv d1 and 8 Etoposide (VP-16) 70 mg/m^2^/d iv d1-3 Procarbazine 60 mg/m^2^/d po d1-10 Prednisone 60 mg/m^2^/d po d1-10 Q4w × 6 cycles
**Chlorambucil** 10 mg/m^2^ PO day 1 – day 7 Q4w × 12 cycles
**CEOP ± R**
Rituximab 375 mg/m^**2**^ iv d1 Cyclophophamide 750 mg/m^**2**^ iv d1 Etoposide 50 mg/m^**2**^ iv d1 followed by 100 mg oral on d2 and d3. Vincristine 1.4 mg/m^**2**^ (max 2 mg) iv d1 Prednisone 100 mg po qd d1-5 Q3w × 6 cycles
**CHOP ± R**
Rituximab 375 mg/m^2^ iv d1 Cyclophophamide 750 mg/m^2^ iv d1 Doxorubicin (Adriamycin) 50 mg/m^2^ iv d1 Vincristine 1.4 mg/m^2^ (max 2 mg) iv d1 Prednisone 100 mg po qd d1-5 Q3w × 6 cycles
**CODOX-M** (Modified for low risk patients: single extra-abdominal mass or completely resected abdominal mass and normal serum LDH)
Cyclophosphamide 800 mg/m^**2**^ iv d1 Cyclophosphamide 200 mg/m^**2**^/d iv d2-5 Doxorubicin (Adriamycin) 40 mg/m^**2**^ iv d1 Vincristine 1.5 mg/m^**2**^ iv d1, 8 Methotrexate (MTX) 1200 mg/m^**2**^ iv over 1 h d10, then 240 mg/m^**2**^ per hour civi for the next 23 h Leucovorin 50 mg iv q6 h begins 36 h from the start of MTX till MTX level < 0.05 uM G-CSF begins 24 h from the start of leucovorin till ANC > 1000/mL **CNS prophylaxis**: Intrathecal Cytarabine (Ara-C) 70 mg d1, Methotrexate (Mtx) 12 mg d3 Total of 3 cycles
**CODOX-M/IVAC** (for high risk patients: do not meet low risk criteria)
**Cycle 1 and 3** (CODOX-M) Cyclophosphamide 800 mg/m^2^ iv d1 Cyclophosphamide 200 mg/m^2^d iv d2-5 Doxorubicin (Adriamycin) 40 mg/m^2^ iv d1 Vincristine 1.5 mg/m^2^ iv d1, 8 for cycle 1 and d1, 8, 15 for cycle 3 Methotrexate (MTX) 1200 mg/m^2^ iv over 1 h d10, then 240 mg/m^2^ per hour civi for the next 23 h Leucovorin 50 mg iv q6 h begins 36 h from the start of MTX till MTX level < 0.05 uM G-CSF begins 24 h from the start of Leucovorin till ANC > 1000/mL **CNS prophylaxis:** Intrathecal Cytarabine (Ara-C) 70 mg d1 and 3, Methotrexate (MTX) 12 mg d15 CNS treatment Cycle 1: Intrathecal Cytarabine (Ara-C) 70 mg d1, 3 and 5, Methotrexate (MTX) 12 mg d15 and 17 Cycle 3: Intrathecal Cytarabine (Ara-C) 70 mg d1 and 3, Methotrexate (MTX) 12 mg d15 **Cycle 2 and 4** (IVAC) Ifosfamide 1500 mg/m^2^/d iv d1-5 Etoposide (VP-16) 60 mg/m^2^/d iv d1-5 Cytarabine (Ara-C) 2000 mg/m^2^ iv q12 h d1 and 2 (total 4 doses) G-CSF begins 24 h after completion of chemotherapy till ANC > 1000/mL **CNS prophylaxis:** Intrathecal Methotrexate (MTX) 12 mg d5 **CNS treatment:** Cycle 2: Intrathecal Methotrexate (MTX) 12 mg d5, Cytarabine (Ara-C) 70 mg d7 and 9 Cycle 4: Intrathecal Methotrexate (MTX) 12 mg d5 **Radiotherapy for CNS disease and testicular involvement**
**COPP**
Cyclophosphamide 600 mg/m^2^ iv d1 and 8 Vincristine 1.4 mg/m^**2**^ (max 2 mg) iv d1 and 8 Procarbazine 60 mg/m^**2**^/d po d1-10 Prednisone 60 mg/m^**2**^/d po d1-10 Q4w × 6 cycles
**CVP ± R**
Rituximab 375 mg/m^**2**^ iv d1 Cyclophophamide 750 mg/m^**2**^ iv d1 Vincristine 1.4 mg/m^2^ (max 2 mg) iv d1 Prednisone 100 mg po qd d1-5 Q3w × 6–8 cycles
**DHAP ± R**
Rituximab 375 mg/m^**2**^ iv d1 Dexamethasone (Decadron) 40 mg po qd d1-4 Cisplatin 100 mg/m^**2**^ iv over 24 h d1 Cytarabine (Ara-C) 2000 mg/m^**2**^ iv q12 h for 2 doses d2 Q3-4w
**EPOCH ± R**
Rituximab 375 mg/m^**2**^ iv d1 Etoposide (VP-16) 50 mg/m^**2**^/d civi d1-4 Prednisone 60 mg/m^**2**^/d po d1-5 Vincristine 0.4 mg/m^**2**^/d civi d1-4 Doxorubicin (Adriamycin) 10 mg/m^**2**^/d civi d1-4 Cyclophosphamide 750 mg/m^**2**^ iv over 15 min d5 G-CSF 5 µg/kg sc qd beginining on d6 till ANC > 10,000/uL Q3w × 6–8 cycles
**EPOCH-Dose-adjusted ± R**
Rituximab 375 mg/m^**2**^ iv d1 Etoposide (VP-16) 50 mg/m^**2**^/d civi d1-4 Prednisone 60 mg/m^**2**^**/**d po d1-5 Vincristine 0.4 mg/m^**2**^/d civi d1-4 Doxorubicin (Adriamycin) 10 mg/m^**2**^/d civi d1-4 Cyclophosphamide 750 mg/m^**2**^ iv over 15 min d5 Bactrim DS 1 tablet po bid tiw G-CSF 5 µg/kg scqd beginining on d6 till ANC > 5000/uL Q3w × 6–8 cycles Dose-adjustment paradigm based on twice weekly CBC (dose adjustment above starting doses apply to Etoposide (VP-16), Doxorubicin (Adriamycin) and Cyclophosphamide If nadir ANC > 500/uL, 20% increase in Etoposide (VP-16), Doxorubicin (Adriamycin) and Cyclophosphamide above last cycle If nadir ANC < 500/uL on 1 or 2 measurements, same doses as last cycle If nadir ANC < 500/uL on at least 3 measurements, or nadir platelet < 25,000/uL on 1 measurement, 20% decrease in Etoposide (VP-16), Doxorubicin (Adriamycin) and Cyclophosphamide below last cycle
**ESHAP ± R**
Rituximab 375 mg/m^**2**^ iv d1 Etoposide (VP-16) 40 mg/m^**2**^/d iv over 1 h d1-4 Methylprednisolone 500 mg/d iv over 15 min d1-5 Cisplatin 25 mg/m^**2**^/d civi d1-4 Cytarabine (Ara-C) 2000 mg/m^**2**^ iv over 2 h d5 Q3-4w × 6–8 cycles
**FC ± R**
Rituximab 375 mg/m^**2**^ iv d0, cycle 1 Rituximab 500 mg/m^**2**^ iv d1, cycle 2 – 6 Fludarabine 25 mg/m^**2**^ iv d1–d3 Cyclophsphamide 250 mg/m^**2**^ po d1–d3 Q 4w × 6 cycles
**GDP ± R**
Rituximab 375 mg/m^**2**^ iv d1 Gemcitabine 1000 mg/m^**2**^ iv d1 and d 8 Dexamethasone 40 mg po qd d1-4 Cisplatin 100 mg/m^**2**^ iv over 24 h Q 3w
**GMALL-B-ALL/NHL 2002**
**Prephase → A1 → B1 → C1 → A2 → B2 → C2** Cyclophosphamide 200 mg/m^2^/d iv d1-5 Prednisone 60 mg/m^2^/d p o d1-7 **Cycle A1 on day** 7 [Repeat A2 on Day 77] Rituximab 375 mg/m^2^** iv d7** Dexamethasone (Decadron) 10 mg/m^2^/d po d7-12 Vincristine 2 mg iv d7 Ifosfamide 800 mg/m^2^/d iv over 1 h d8-12 Mesna 200 mg/m^2^ iv at 0, 4 and 8 h after ifosfamide d8-12 Methotrexate (MTX) 150 mg/m^2^ iv over 30 min d1, followed by 1350 mg/m^2^ civi over 23.5 h on d8 Leucovorin 50 mg/m^2^ iv 36 h after start of MTX, followed by 15 mg/m^2^ iv q 6 h till MTX level < 0.05 uM Cytarabine (Ara-c) 150 mg/m^2^/d civi d 11 and 12 Etoposide (VP-16) 80 mg/m^2^/d iv over 1 h d 11 and 12 Prophylaxis Triple IT-Day 8 (12) GCSF 5 µg/kg S/C from d14 onwards till ANC recovery to > 500/cmm **Cycle B1 on day 28** [Repeat B2 on Day 98] Rituximab 375 mg/m^2^** iv d28** Dexamethasone (Decadron) 10 mg/m^2^/d po d29-33 Vincristine 2 mg iv d29 Cyclophosphamide 200 mg/m^2^/d iv d29-33 Methotrexate (MTX) 150 mg/m^2^ iv over 30 min d1, followed by 1350 mg/m^2^ civi over 23.5 h on d29 Leucovorin 50 mg/m^2^ iv 36 h after start of MTX, followed by 15 mg/m^2^ iv q6 h till MTX level < 0.05 uM Doxorubicin (Adriamycin) 25 mg/m^2^/d iv bolus d32 and 33 Prophylaxis Triple IT-Day 29 (33) GCSF 5 µg/kg S/C from d35 onwards…… till ANC recovery to > 500/cmm **Cycle C1 on day 49** [Repeat C2 on Day 119] Rituximab 375 mg/m^2^** iv d49** Dexamethasone (Decadron) 10 mg/m^2^/d po d50-54 Vindesin 3 mg/m^2^ iv d50 Methotrexate (MTX) 150 mg/m^2^ iv over 30 min d1, followed by 1350 mg/m^2^ civi over 23.5 h on d50 Leucovorin 50 mg/m^2^ iv 36 h after start of MTX, followed by 15 mg/m^2^ iv q6 h till MTX level < 0.05 uM Etoposide (VP-16) 250 mg/m^2^/d iv over 1 h d 53 and 54 HD Cytarabine 2 × 2 gm/m^2^** ci 3 h every 12 h d54** Prophylaxis Triple IT-Day 49 (119) GCSF 5 µg/Kg S/C from d56 onwards till ANC recovery to > 500/cmm
**GemOx ± R**
Rituximab 375 mg/m^**2**^ iv d1 Gemcitabine 1000 mg/m^**2**^ iv d2 Oxaliplatin 100 mg/m^**2**^ iv over 2 h d2 Q2-3w
**Hyper-CVAD/MTX-Ara-C**
**Cycle 1, 3, 5, 7** (3–4 weeks/cycle) Cyclophosphamide 300 mg/m^**2**^ iv over 2 h q12 h × 6 doses d1-3 Mesna 600 mg/m^**2**^/d civi d1-3 to start 1 h before cyclophosphamide till 12 h after completion of cyclophosphamide Vincristine 2 mg iv d4, 11 Doxorubicin (Adriamycin) 50 mg/m^**2**^iv over 24 h (over 48 h if LVEF < 50%) d4 Dexamethasone (Decadron) 40 mg po or iv qd d1-4 and d11-14 **Cycle 2, 4, 6, 8** (3–4 weeks/cycle) Methotrexate (MTX) 200 mg/m^**2**^iv over 2 h followed by 800 mg/m^2^ civi over 22 h d1 Cytarabine (Ara-C) 3 g/m^**2**^ (1 g/m^**2**^ for patients > 60 years old) iv over 2 h q12 h × 4 doses d2-3 Leucovorin 50 mg iv q6 h starting 12 h after completion of MTX till MTX level < 0.05 uM **Intrathecal chemotherapy** *Prophylaxis* Methotrexate (MTX) 12 mg d2 of each cycle for a total of 3–4 treatments Cytarabine (Ara-C) 100 mg d8 of each cycle for a total of 3–4 treatments *Therapeutic* Intrathecal chemotherapy twice a week (Methotrexate (MTX) 12 mg and Cytarabine (Ara-C) 100 mg respectively) till no more cancer cells in CSF, then decrease intrathecal chemotherapy to once a week × 4, followed by Methotrexate (MTX) 12 mg d2, Cytarabine (Ara-C) 100 mg d8 for the remaining chemotherapy cycles **Cranial radiotherapy 24–30 Gy if cranial nerve palsies**
**Ibrutinib** 420 mg PO daily till toxicity or progression
**ICE ± R**
Rituximab 375 mg/m^**2**^ iv d1 Ifosfamide 5000 mg/m^**2**^ mixed with Mesna 5000 mg/m^2^ iv over 24 h d2 Carboplatin AUC 5 (max 800 mg) iv d2 Etoposide (VP-16) 100 mg/m^2^/d iv d1-3 G-CSF 5 µg/kg sc qd d5-12 Q3w × 3 to 6 cycles
**MINE ± R**
Rituximab 375 mg/m^**2**^ iv d1 Mesna 1330 mg/M^**2**^/d iv over 1 h with ifosfamide d1-3, then 500 mg po 4 h after ifosfamide d1-3 Ifosfamide 1330 mg/M^**2**^/d iv over 1 h d1-3 Mitoxantrone 8 mg/M^**2**^ iv d1 Etoposide (VP-16) 65 mg/M^**2**^/d iv over 1 h d1-3 Q3w × 3–6 cycles
**Methotrexate-High Dose for Primary CNS Lymphoma**
Methotrexate (MTX) 8 g/m^**2**^iv over 4 h q2w till CR or up to 8 cycles, followed by 8 gm/m^**2**^ iv qm × 11 months
**MPV-R + RT + Ara-C**
Rituximab 500 mg/m^**2**^ iv over 5 h d1 of each cycle Methotrexate (MTX) 3.5 g/m^**2**^iv over 2 h d2 of each cycle Leucovorin 20–25 mg q6 h starting 24 h after MTX infusion for 72 h or until serum MTX level < 1 × 10^−8^ mg/dL. Increase leucovorin to 40 mg q4 h if MTX level > 1 × 10^−5^ mg/dL at 48 h or > 1 × 10^−8^ mg/dL at 72 h Vincristine 1.4 mg/m^**2**^ (max 2.8 mg) iv d2 of each cycle Procarbazine 100 mg/m^**2**^ poqd d1-7 of odd-numbered cycles only G-CSF 5 µg/kg/d sc for 3 to 5 days starting 24 h after the last dose of procarbazine during odd-numbered cycles, and starting 96 h after MTX infusion or when MTX levels < 1 × 10^−8^ mg/dL during even-numbered cycles If positive CSF cytology: intra-ommaya Methotrexate (MTX) 12 mg between days 5 and 12 of each cycle Q2w × 5–7cycles Whole-brain radiotherapy (WBRT) 1.8 Gy/d for 13 days to a total of 23.4 Gy beginning 3–5 weeks after the completion of R-MPV Consolidation Cytarabine (Ara-C) 3 g/m^**2**^**/**d (max 6 g) iv over 3 h for 2 days G-CSF 5 µg/kg/d sc for 10 days starting 48 h after completion of Ara-C A second cycle of Cytarabine (Ara-C) is given 1 month later
**R-mini-CHOP**
Rituximab 375 mg/m^**2**^ iv d1 Cyclophophamide 400 mg/m^**2**^ iv d1 Doxorubicin (Adriamycin) 25 mg/m^**2**^ iv d1 Vincristine 1.0 mg iv d1 Prednisone 40 mg/m^**2**^ poqd d1-5 Q3w × 6 cycles
**SMILE Chemotherapy Protoco**l
Methotrexate 2 g/m^**2**^ iv (6 h) on Day 1 Leucovorin 15 mg × 4 iv or po on day 2, 3, 4 Ifosfamide 1500 mg/M^**2**^ iv on day 2, 3, 4 Mesna 300 mg/M^**2**^ × 3 iv on day 2, 3, 4 Dexamethasone 40 mg/d iv or po on day 2, 3, 4 Etoposide 100 mg/M^**2**^ iv on day 2, 3, 4 L-asparaginase (*Escherichia coli*) 6000 U/m^**2**^ iv on day 8, 10, 12, 14, 16, 18, 20 G-CSF SC or iv Day 6 to WBC > 5000/µL Repeat every 28 days.
**Temozolomide SA**
Temozolomide 150 mg/M^**2**^/d po d1-5 q4w till toxicity or progression of disease

## References

[1] Doval DC, Bhurani D, Nair R, Gujral S, Malhotra P, Ramanan G (2017). Indian Council of Medical Research Consensus document for the management of non-Hodgkin’s lymphoma (high grade). Indian J Med Paediatr Oncol.

[2] Willemze R, Hodak E, Zinzani PL, Specht L, Ladetto M (2013). Primary cutaneous lymphomas: ESMO Clinical Practice Guidelines for diagnosis, treatment and follow-up. Ann Oncol.

[3] Nair R, Arora N, Mallath MK (2016). Epidemiology of Non-Hodgkin’s lymphoma in India. Oncology.

[4] Swerdlow SH (2008). WHO classification of tumours of haematopoietic and lymphoid tissues. WHO classification of tumors.

[5] National Institute for Health and Care Excellence (2016) Non-Hodgkin’s lymphoma: diagnosis and management: NICE guideline [NG52] 27466666

[6] Mckay P, Leach M, Jackson R, Cook G, Rule S (2012). Guidelines for the investigation and management of mantle cell lymphoma. Br J Haematol.

[7] Dreyling M, Thieblemont C, Gallamini A, Arcaini L, Campo E, Hermine O (2013). ESMO Consensus conferences: guidelines on malignant lymphoma. Part 2: marginal zone lymphoma, mantle cell lymphoma, peripheral T-cell lymphoma. Ann Oncol.

[8] National Health Services (2017) North west coast strategic clinical networks. Network clinical guidelines and treatment algorithm for primary central nervous system lymphoma (PCNSL) and primary intraocular lymphoma (PIOL)

[9] McNamara C, Davies J, Dyer M, Hoskin P, Illidge T, Lyttelton M (2012). Guidelines on the investigation and management of follicular lymphoma. Br J Haematol.

[10] National Comprehensive Cancer Network (2014) Clinical practice guidelines in oncology (NCCN Guidelines^®^) Non-Hodgkin’s lymphoma. Version 4

[11] Carbone PP, Kaplan HS, Musshoff K, Smithers DW, Tubiana M (1971). Report of the committee on Hodgkin’s disease staging classification. Cancer Res.

[12] Brepoels L, Stroobants S, De Wever W, Spaepen K, Vandenberghe P, Thomas J (2007). Aggressive and indolent non-Hodgkin’s lymphoma: response assessment by integrated international workshop criteria. Leuk Lymphoma.

[13] Cheson BD, Fisher RI, Barrington SF, Cavalli F, Schwartz LH, Zucca E (2014). Recommendations for initial evaluation, staging, and response assessment of Hodgkin and non-Hodgkin lymphoma: the lugano classification. J Clin Oncol.

[14] Mittal BR, Manohar K, Malhotra P, Das R, Kashyap R, Bhattacharya A (2011). Can fluorodeoxyglucose positron emission tomography/computed tomography avoid negative iliac crest biopsies in evaluation of marrow involvement by lymphoma at time of initial staging?. Leuk Lymphoma.

[15] Rai KR, Sawitsky A, Cronkite EP, Chanana AD, Levy RN, Pasternack B (1975). Clinical staging of chronic lymphocytic leukemia. Blood.

[16] Binet JL, Leporrier M, Dighiero G, Charron D, D’Athis P, Vaugier G (1977). A clinical staging system for chronic lymphocytic leukemia: prognostic significance. Cancer.

[17] Oken MM, Creech RH, Tormey DC, Horton J, Davis TE, McFadden ET (1982). Toxicity and response criteria of the Eastern Cooperative Oncology Group. Am J Clin Oncol.

[18] International CLL-IPI Working Group (2016). An international prognostic index for patients with chronic lymphocytic leukaemia (CLL-IPI): a meta-analysis of individual patient data. Lancet Oncol.

[19] Molica S, Shanafelt TD, Giannarelli D, Gentile M, Mirabelli R, Cutrona G (2016). The chronic lymphocytic leukemia international prognostic index predicts time to first treatment in early CLL: independent validation in a prospective cohort of early stage patients. Am J Hematol.

[20] International Non-Hodgkin’s Lymphoma Prognostic Factors Project (1993). A predictive model for aggressive non-Hodgkin’s lymphoma. N Engl J Med.

[21] Sehn LH, Berry B, Chhanabhai M, Fitzgerald C, Gill K, Hoskins P (2007). The revised International Prognostic Index (R-IPI) is a better predictor of outcome than the standard IPI for patients with diffuse large B-cell lymphoma treated with R-CHOP. Blood.

[22] Solal-Celigny P, Roy P, Colombat P, White J, Armitage JO, Arranz-Saez R (2004). Follicular lymphoma international prognostic index. Blood.

[23] Schmitz N, Zeynalova S, Nickelsen M, Ziepert M, Pfreundschuh M, Glass B (2013). A new prognostic model to assess the risk of CNS disease in patients with aggressive B-cell lymphoma. Hematol Oncol.

[24] Böll B, Görgen H, Fuchs M, Pluetschow A, Eich HT, Bargetzi MJ (2013). ABVD in older patients with early-stage Hodgkin lymphoma treated within the German Hodgkin Study Group HD10 and HD11 trials. J Clin Oncol.

[25] Johnson P, Federico M, Kirkwood A, Fossa A, Berkahn L, Carella A (2016). Adapted treatment guided by interim PET-CT scan in advanced Hodgkin’s lymphoma. N Engl J Med.

[26] Uhm J, Kuruvilla J (2012). Treatment of newly diagnosed advanced stage Hodgkin lymphoma. Blood Rev.

[27] Aleman BM, Raemaekers JM, Tomiŝiĉ R, Baaijens MH, Bortolus R, Lybeert ML (2007). Involved-field radiotherapy for patients in partial remission after chemotherapy for advanced Hodgkin’s lymphoma. Int J Radiat Oncol Biol Phys.

[28] Ganesan P, Kumar L, Raina V, Sharma A, Bakhshi S, Sreenivas V (2011). Hodgkin’s lymphoma—long-term outcome: an experience from a tertiary care cancer center in North India. Ann Hematol.

[29] Jain H, Sengar M, Nair R, Menon H, Laskar S, Shet T (2015). Treatment results in advanced stage Hodgkin’s lymphoma: a retrospective study. J Postgrad Med.

[30] Laskar S, Gupta T, Vimal S, Muckaden M, Saikia T, Pai S (2004). Consolidation radiation after complete remission in Hodgkin’s disease following six cycles of doxorubicin, bleomycin, vinblastine, and dacarbazine chemotherapy: is there a need?. J Clin Oncol.

[31] Connors JM, Jurczak W, Straus DJ, Ansell SM, Kim WS, Gallamini A (2018). Brentuximab vedotin with chemotherapy for stage III or IV Hodgkin’s lymphoma. N Engl J Med.

[32] Crump M, Herst J, Baldassarre F, Sussman J, MacEachern J, Hodgson D (2015). Evidence-based focused review of the role of radiation therapy in the treatment of early-stage Hodgkin lymphoma. Blood.

[33] Eich HT, Diehl V, Görgen H, Pabst T, Markova J, Debus J (2010). Intensified chemotherapy and dose-reduced involved-field radiotherapy in patients with early unfavorable Hodgkin’s lymphoma: final analysis of the German Hodgkin Study Group HD11 trial. J Clin Oncol.

[34] Engert A, Haverkamp H, Kobe C, Markova J, Renner C, Ho A (2012). Reduced-intensity chemotherapy and PET-guided radiotherapy in patients with advanced stage Hodgkin’s lymphoma (HD15 trial): a randomised, open-label, phase 3 non-inferiority trial. Lancet.

[35] von Tresckow B, Plutschow A, Fuchs M, Klimm B, Markova J, Lohri A (2012). Dose-intensification in early unfavorable Hodgkin’s lymphoma: final analysis of the German Hodgkin Study Group HD14 trial. J Clin Oncol.

[36] Connors JM, Ansell SM, Fanale M, Park SI, Younes A (2017). Five-year follow-up of brentuximab vedotin combined with ABVD or AVD for advanced-stage classical Hodgkin lymphoma. Blood.

[37] Federico M, Luminari S, Dondi A, Tucci A, Vitolo U, Rigacci L (2013). R-CVP versus R-CHOP versus R-FM for the initial treatment of patients with advanced-stage follicular lymphoma: results of the FOLL05 trial conducted by the Fondazione Italiana Linfomi. J Clin Oncol.

[38] Gogia A, Raina V, Kumar L, Sharma A, Sharma MC, Mallick SR (2017). Follicular lymphoma: an Institutional Analysis. Asian Pac J Cancer Prev.

[39] Hainsworth JD, Litchy S, Burris HA, Scullin DC, Corso SW, Yardley DA (2002). Rituximab as first-line and maintenance therapy for patients with indolent non-Hodgkin’s lymphoma. J Clin Oncol.

[40] Mac Manus MP, Hoppe RT (1996). Is radiotherapy curative for stage I and II low-grade follicular lymphoma? Results of a long-term follow-up study of patients treated at Stanford University. J Clin Oncol.

[41] Marcus R, Imrie K, Solal-Celigny P, Catalano JV, Dmoszynska A, Raposo JC (2008). Phase III study of R-CVP compared with cyclophosphamide, vincristine, and prednisone alone in patients with previously untreated advanced follicular lymphoma. J Clin Oncol.

[42] Rummel MJ, Niederle N, Maschmeyer G, Banat GA, von Grunhagen U, Losem C (2013). Bendamustine plus rituximab versus CHOP plus rituximab as first-line treatment for patients with indolent and mantle-cell lymphomas: an open-label, multicentre, randomised, phase 3 non-inferiority trial. Lancet.

[43] Schulz H, Bohlius JF, Trelle S, Skoetz N, Reiser M, Kober T (2007). Immunochemotherapy with rituximab and overall survival in patients with indolent or mantle cell lymphoma: a systematic review and meta-analysis. J Natl Cancer Inst.

[44] Eichhorst B, Fink AM, Bahlo J, Busch R, Kovacs G, Maurer C (2016). First-line chemoimmunotherapy with bendamustine and rituximab versus fludarabine, cyclophosphamide, and rituximab in patients with advanced chronic lymphocytic leukaemia (CLL10): an international, open-label, randomised, phase 3, non-inferiority trial. Lancet Oncol.

[45] Lobetti-Bodoni C, Bertoni F, Stussi G, Cavalli F, Zucca E (2013). The changing paradigm of chronic lymphocytic leukemia management. Eur J Intern Med.

[46] Byrd JC, Furman RR, Coutre SE, Burger JA, Blum KA, Coleman M (2015). Three-year follow-up of treatment-naive and previously treated patients with CLL and SLL receiving single-agent ibrutinib. Blood.

[47] Gogia A, Sharma A, Raina V, Kumar L, Vishnubhatla S, Gupta R (2012). Assessment of 285 cases of chronic lymphocytic leukemia seen at single large tertiary center in Northern India. Leuk Lymphoma.

[48] Catovsky D, Else M, Richards S (2011). Chlorambucil—still not bad: a reappraisal. Clin Lymphoma Myeloma Leuk.

[49] Miller TP, Dahlberg S, Cassady JR, Adelstein DJ, Spier CM, Grogan TM (1998). Chemotherapy alone compared with chemotherapy plus radiotherapy for localized intermediate-and high-grade non-Hodgkin’s lymphoma. N Engl J Med.

[50] Peyrade F, Jardin F, Thieblemont C, Thyss A, Emile J-F, Castaigne S (2011). Attenuated immunochemotherapy regimen (R-miniCHOP) in elderly patients older than 80 years with diffuse large B-cell lymphoma: a multicentre, single-arm, phase 2 trial. Lancet Oncol.

[51] Prakash G, Sharma A, Raina V, Kumar L, Sharma M, Mohanti B (2012). B cell non-Hodgkin’s lymphoma: experience from a tertiary care cancer center. Ann Hematol.

[52] Dunleavy K, Pittaluga S, Maeda LS, Advani R, Chen CC, Hessler J (2013). Dose-adjusted EPOCH-rituximab therapy in primary mediastinal B-cell lymphoma. N Engl J Med.

[53] Roy PS, John S, Karankal S, Kannan S, Pawaskar P, Gawande J (2013). Comparison of the efficacy and safety of Rituximab (Mabthera™) and its biosimilar (Reditux™) in diffuse large B-cell lymphoma patients treated with chemo-immunotherapy: a retrospective analysis. Indian J Med Paediatr Oncol.

[54] Persky DO, Unger JM, Spier CM, Stea B, LeBlanc M, McCarty MJ (2008). Phase II study of rituximab plus three cycles of CHOP and involved-field radiotherapy for patients with limited-stage aggressive B-cell lymphoma: Southwest Oncology Group study 0014. J Clin Oncol.

[55] Nimmagadda RB, Digumarti R, Nair R, Bhurani D, Raina V, Aggarwal S (2013). Histopathological pattern of lymphomas and clinical presentation and outcomes of diffuse large B cell lymphoma: a multicenter registry based study from India. Indian J Med Paediatr Oncol.

[56] Kluin-Nelemans H, Hoster E, Hermine O, Walewski J, Trneny M, Geisler C (2012). Treatment of older patients with mantle-cell lymphoma. N Engl J Med.

[57] Delarue R, Haioun C, Ribrag V, Brice P, Delmer A, Tilly H (2013). CHOP and DHAP plus rituximab followed by autologous stem cell transplantation in mantle cell lymphoma: a phase 2 study from the Groupe d’Etude des Lymphomes de l’Adulte. Blood.

[58] Campo E, Rule S (2015). Mantle cell lymphoma: evolving management strategies. Blood.

[59] Das CK, Gogia A, Kumar L, Sharma A, Sharma MC, Mallick SR (2016). Mantle cell lymphoma: a North Indian Tertiary Care Centre experience. Asian Pac J Cancer Prev.

[60] Dunleavy K, Pittaluga S, Shovlin M, Steinberg SM, Cole D, Grant C (2013). Low-intensity therapy in adults with Burkitt’s lymphoma. N Engl J Med.

[61] Hoelzer D, Walewski J, Döhner H, Viardot A, Hiddemann W, Spiekermann K (2014). Improved outcome of adult Burkitt lymphoma/leukemia with rituximab and chemotherapy: report of a large prospective multicenter trial. Blood.

[62] Gale J, Simmonds PD, Mead GM, Sweetenham JW, Wright DH (2000). Enteropathy-type intestinal T-cell lymphoma: clinical features and treatment of 31 patients in a single center. J Clin Oncol.

[63] Besson C, Panelatti G, Delaunay C, Gonin C, Brebion A, Hermine O (2002). Treatment of adult T-cell leukemia-lymphoma by CHOP followed by therapy with antinucleosides, alpha interferon and oral etoposide. Leuk Lymphoma.

[64] Battiwalla M, Melenhorst J, Saunthararajah Y, Nakamura R, Molldrem J, Young NS (2003). HLA-DR4 predicts haematological response to cyclosporine in T-large granular lymphocyte lymphoproliferative disorders. Br J Haematol.

[65] Belhadj K, Reyes F, Farcet J-P, Tilly H, Bastard C, Angonin R (2003). Hepatosplenic γδ T-cell lymphoma is a rare clinicopathologic entity with poor outcome: report on a series of 21 patients. Blood.

[66] Fukushima T, Miyazaki Y, Honda S, Kawano F, Moriuchi Y, Masuda M (2005). Allogeneic hematopoietic stem cell transplantation provides sustained long-term survival for patients with adult T-cell leukemia/lymphoma. Leukemia.

[67] Advani R, Horwitz S, Zelenetz A, Horning SJ (2007). Angioimmunoblastic T cell lymphoma: treatment experience with cyclosporine. Leuk Lymphoma.

[68] Bishton MJ, Haynes AP (2007). Combination chemotherapy followed by autologous stem cell transplant for enteropathy-associated T cell lymphoma. Br J Haematol.

[69] Jaccard A, Petit B, Girault S, Suarez F, Gressin R, Zini J-M (2009). L-asparaginase-based treatment of 15 western patients with extranodal NK/T-cell lymphoma and leukemia and a review of the literature. Ann Oncol.

[70] Czyz A, Romejko-Jarosinska J, Helbig G, Knopinska-Posluszny W, Poplawska L, Piatkowska-Jakubas B (2013). Autologous stem cell transplantation as consolidation therapy for patients with peripheral T cell lymphoma in first remission: long-term outcome and risk factors analysis. Ann Hematol.

[71] Nair RA, Jacob PM, Nair SG, Prem S, Jayasudha AV, Sindhu NP (2013). Adult T cell leukaemia/lymphoma in Kerala, South India: are we staring at the tip of the iceberg?. J Hematop.

[72] Olsen EA (2003). Interferon in the treatment of cutaneous T-cell lymphoma. Dermatol Ther.

[73] Prince HM, Kim YH, Horwitz SM, Dummer R, Scarisbrick J, Quaglino P (2017). Brentuximab vedotin or physician’s choice in CD30-positive cutaneous T-cell lymphoma (ALCANZA): an international, open-label, randomised, phase 3, multicentre trial. Lancet.

[74] Zhang C, Duvic M (2006). Treatment of cutaneous T-cell lymphoma with retinoids. Dermatol Ther.

[75] Parrilla Castellar ER, Jaffe ES, Said JW, Swerdlow SH, Ketterling RP, Knudson RA (2014). ALK-negative anaplastic large cell lymphoma is a genetically heterogeneous disease with widely disparate clinical outcomes. Blood.

[76] Grommes C, DeAngelis LM (2017). Primary CNS lymphoma. J Clin Oncol.

[77] Witzig TE, Geyer SM, Kurtin PJ, Colgan JP, Inwards DJ, Micallef INM (2008). Salvage chemotherapy with rituximab DHAP for relapsed non-Hodgkin lymphoma: a phase II trial in the North Central Cancer Treatment Group. Leuk Lymphoma.

[78] Velasquez WS, McLaughlin P, Tucker S, Hagemeister FB, Swan F, Rodriguez MA (1994). ESHAP—an effective chemotherapy regimen in refractory and relapsing lymphoma: a 4-year follow-up study. J Clin Oncol.

[79] Kewalramani T, Zelenetz AD, Nimer SD, Portlock C, Straus D, Noy A (2004). Rituximab and ICE as second-line therapy before autologous stem cell transplantation for relapsed or primary refractory diffuse large B-cell lymphoma. Blood.

[80] Baetz T, Belch A, Couban S, Imrie K, Yau J, Myers R (2003). Gemcitabine, dexamethasone and cisplatin is an active and non-toxic chemotherapy regimen in relapsed or refractory Hodgkin’s disease: a phase II study by the National Cancer Institute of Canada Clinical Trials Group. Ann Oncol.

[81] Gutierrez A, Rodriguez J, Martinez-Serra J, Gines J, Paredes P, Garcia F (2014). Gemcitabine and oxaliplatinum: an effective regimen in patients with refractory and relapsing Hodgkin lymphoma. Onco Targets Ther.

[82] Ferme C, Bastion Y, Lepage E, Berger F, Brice P, Morel P (1995). The MINE regimen as intensive salvage chemotherapy for relapsed and refractory Hodgkin’s disease. Ann Oncol.

[83] Villa D, Seshadri T, Puig N, Massey C, Tsang R, Keating A (2012). Second-line salvage chemotherapy for transplant-eligible patients with Hodgkin’s lymphoma resistant to platinum-containing first-line salvage chemotherapy. Haematologica.

[84] Seyfarth B, Josting A, Dreyling M, Schmitz N (2006). Relapse in common lymphoma subtypes: salvage treatment options for follicular lymphoma, diffuse large cell lymphoma and Hodgkin disease. Br J Haematol.

[85] Mocikova H, Sykorova A, Stepankova P, Markova J, Michalka J, Kral Z (2014). Treatment and prognosis of relapsed or refractory Hodgkin lymphoma patients ineligible for stem cell transplantation. Klin Onkol.

[86] Raut LS, Chakrabarti PP (2014). Management of relapsed-refractory diffuse large B cell lymphoma. South Asian J Cancer.

[87] Kuruvilla J, Keating A, Crump M (2011). How I treat relapsed and refractory Hodgkin lymphoma. Blood.

[88] Chao NJ, Rosenberg SA, Horning SJ (1990). CEPP(B): an effective and well-tolerated regimen in poor-risk, aggressive non-Hodgkin’s lymphoma. Blood.

[89] Wang M, Fowler N, Wagner-Bartak N, Feng L, Romaguera J, Neelapu SS (2013). Oral lenalidomide with rituximab in relapsed or refractory diffuse large cell, follicular and transformed lymphoma: a phase II clinical trial. Leukemia.

[90] Corazzelli G, Capobianco G, Arcamone M, Ballerini PF, Iannitto E, Russo F (2009). Long-term results of gemcitabine plus oxaliplatin with and without rituximab as salvage treatment for transplant-ineligible patients with refractory/relapsing B-cell lymphoma. Cancer Chemother Pharmacol.

[91] Gopal AK, Press OW, Shustov AR, Petersdorf SH, Gooley TA, Daniels JT (2010). Efficacy and safety of gemcitabine, carboplatin, dexamethasone, and rituximab in patients with relapsed/refractory lymphoma: a prospective multi-center phase II study by the Puget Sound Oncology Consortium. Leuk Lymphoma.

[92] Jermann M, Jost LM, Taverna C, Jacky E, Honegger HP, Betticher DC (2004). Rituximab-EPOCH, an effective salvage therapy for relapsed, refractory or transformed B-cell lymphomas: results of a phase II study. Ann Oncol.

